# Strain-level heterogeneity in *Vibrio parahaemolyticus* limits the predictive value of baseline planktonic gene expression for surface-associated phenotypes

**DOI:** 10.1038/s41598-026-64000-1

**Published:** 2026-07-27

**Authors:** Xia Huang, Thomas Alter, Roswitha Merle, Vanessa Szott

**Affiliations:** 1 Institute of Food Safety and Food Hygiene, Freie Universität Berlin, Königsweg 69, 14163 Berlin, Germany; 2 Institute of Veterinary Epidemiology and Biostatistics, Freie Universität Berlin, Königsweg 67, 14163 Berlin, Germany; 3 Institute for Animal Hygiene and Environmental Health, Freie Universität Berlin, Robert-von-Ostertag-Str. 8, 14163 Berlin, Germany

**Keywords:** *Vibrio parahaemolyticus*, Strain-level heterogeneity, Gene expression, Surface-associated phenotypes, Predictive limitation, Computational biology and bioinformatics, Genetics, Microbiology

## Abstract

*Vibrio (V.) parahaemolyticus* is a ubiquitous marine bacterium that persists in dynamic marine environments through adaptive behaviors including motility and biofilm formation. Gene expression data obtained under planktonic conditions are widely used to infer surface-associated phenotypes, implicitly assuming that such measurements are informative of these behaviors. However, the extent to which this assumption holds across genetically diverse strains remains unclear. This study systematically assessed whether baseline transcriptional profiles provide predictive value for surface-associated phenotypes across a diverse panel of *V. parahaemolyticus* strains. Phenotypic traits and gene expression patterns varied substantially across individual strains. Correlation analyses revealed no robust associations between transcriptional levels and phenotypes after multiple-testing correction, and predictive modeling further demonstrated that transcriptional profiles do not reliably predict surface-associated phenotypes across strains (leave-one-out cross-validation R^2^ < 0). Principal component analysis (PCA) further confirmed strong strain-dependent transcriptional variation, with no distinct clustering among strains (PC1 and PC2 explaining 44.5% of total variance). These findings demonstrate that strain-level heterogeneity dominates both phenotypic and transcriptional variation and limits the use of baseline planktonic transcription as a predictor of surface-associated traits, highlighting the need to account for strain-level heterogeneity and condition-specific regulation in predictive frameworks.

**Supplementary Information:**

The online version contains supplementary material available at 10.1038/s41598-026-64000-1.

## Introduction

*Vibrio (V.) parahaemolyticus* is a ubiquitous marine bacterium inhabiting coastal and estuarine environments and is a leading food-associated pathogen linked to seafood consumption^1–3^. In natural habitats, the organism encounters rapidly changing physicochemical conditions and transitions between free-living planktonic growth and surface-associated colonization^4–7^. These states differ not only in cellular behavior but also in regulatory activity, as surface contact induces coordinated changes in motility, adhesion, extracellular polysaccharide (EPS) production and quorum-sensing (QS)–dependent gene expression^8,9^.

These phenotypes have been extensively studied, but most investigations have focused on a limited number of strains^10–13^. Such approaches assume that the behavior of individual isolates reflects characteristics of the species. However, bacterial populations frequently exhibit substantial genetic and phenotypic heterogeneity across strains. Such strain-level variability influences regulatory traits, including gene expression patterns, and may therefore affect the interpretability of relationships between transcription and phenotype^14,15^, thereby limiting the generalizability of such approaches.

While differences between planktonic and surface-associated states are well established, it remains unclear whether baseline transcriptional profiles are informative across genetically diverse strains. Transcriptional analyses are widely used to interpret regulatory mechanisms underlying phenotypes such as motility, adhesion and biofilm formation, often based on measurements obtained under standardized planktonic laboratory conditions to allow reproducible and controlled comparisons^14,16–23^. This common practice assumes that transcriptional profiles obtained under planktonic conditions are informative of regulatory states relevant to surface-associated phenotypes. However, this relationship has rarely been systematically evaluated across diverse strain collections. Transcriptomic analyses of *V. parahaemolyticus* during biofilm development have demonstrated extensive regulatory reprogramming, indicating that biofilm formation represents a distinct physiological state rather than a continuation of planktonic growth^24^. As these phenotypes emerge during surface interaction, conditions that differ from the homogeneous liquid cultures in which transcription is typically measured^25–27^, the extent to which transcriptional measurements obtained from planktonic cultures account for strain-level phenotypic variability observed under surface-associated conditions remains insufficiently resolved^28^. This limitation is particularly relevant in the context of strain-level heterogeneity, which may obscure generalizable relationships between transcriptional states and phenotypic outcomes across strains.

Motility and biofilm formation are suitable phenotypic traits for addressing this question, as both are surface-associated behaviors that depend on coordinated regulatory networks^21,29,30^. Notably, *V. parahaemolyticus* possesses a dual flagellar system comprising polar and lateral flagella: the polar flagellum mediates swimming in liquid environments, whereas the lateral flagella are induced upon surface contact to enable swarming motility^31^. In addition to motility, colonization involves adhesion proteins^32^, Type VI Secretion System 2 (T6SS2)^12^, EPS^33^ production, chitinase factors^34^ and other membrane-associated proteins^35,36^, which are regulated by QS systems^33^ and cyclic di-GMP (c-di-GMP) signaling^37,38^.

Therefore, the objective of this study was to systematically evaluate whether baseline transcriptional states measured under standardized planktonic conditions provide predictive value for surface-associated phenotypes across a diverse strain panel. To address this, strain-resolved phenotypic traits (swimming, swarming and biofilm formation) were analyzed alongside targeted gene-content screening and transcriptional profiling across a broad panel of 35 motility-, biofilm- and virulence-associated genes. Phenotypic assays were conducted at temperatures relevant to marine environments and the human host (25 °C, 30 °C and 37 °C), whereas transcriptional profiling was performed under standardized planktonic growth conditions at 30 °C to enable comparison of baseline gene expression. This design enabled direct evaluation of whether transcriptional measurements obtained under planktonic conditions predict surface-associated phenotypic variation across strains.

## Results

**Fig. 1:**
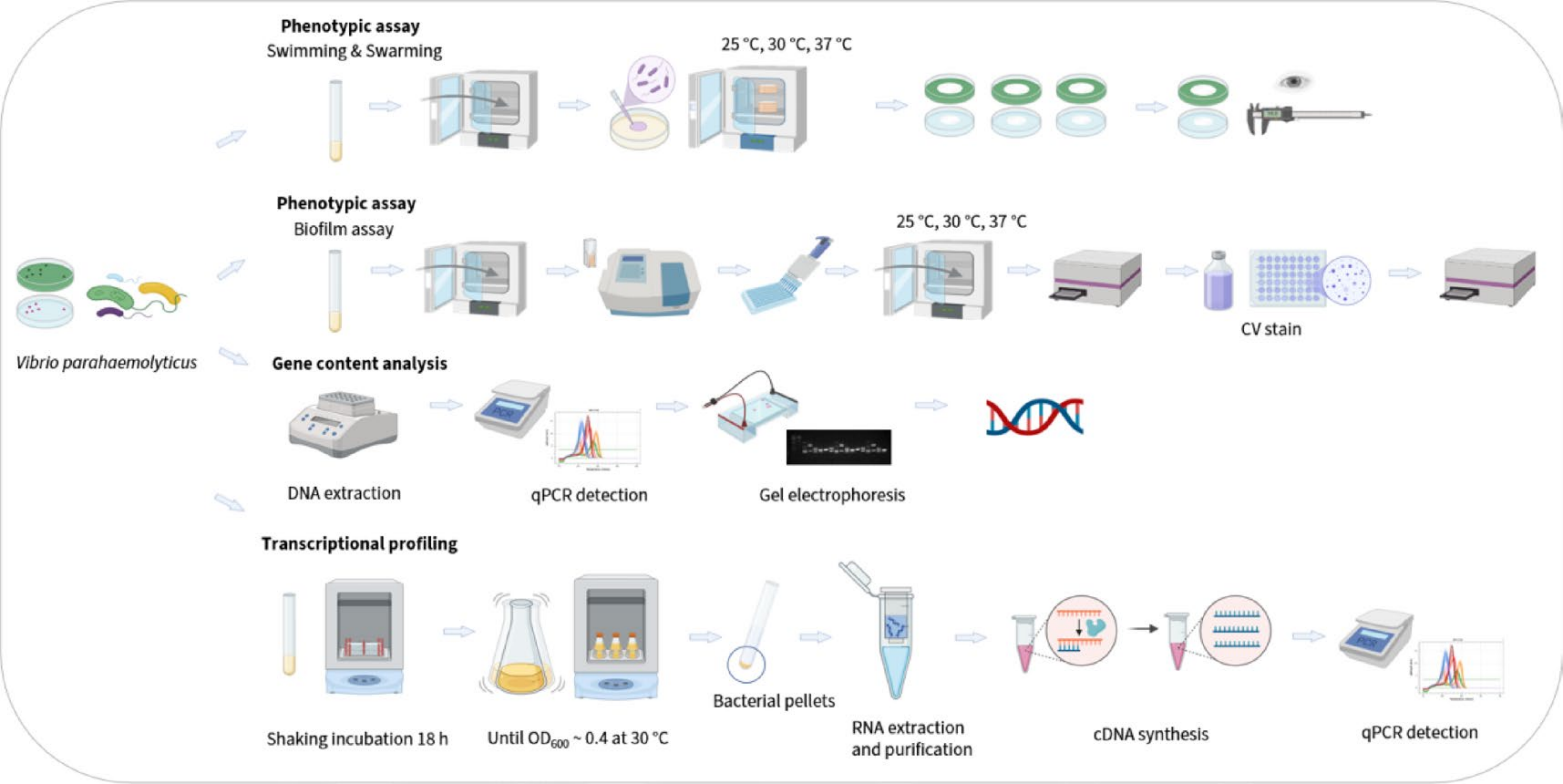
Flow diagram for the phenotypic and genotypic studies. Phenotypic assays included swimming, swarming, and biofilm formation assays conducted at 25 °C, 30 °C, and 37 °C. Gene content analysis and transcriptional profiling were performed to characterize strain-level differences. The figure was created using BioRender.com.

An overview of the experimental design and analytical workflow is shown in Fig. 1.

### Motility (swimming)

Intraclass correlation coefficient (ICC) analysis, derived from linear mixed-effects models, showed substantial strain heterogeneity in swimming behavior, with 55.4% of the total variance in swimming behavior attributable to variance between strains (adjusted ICC = 0.554). After accounting for the fixed effects of temperature and incubation time, the conditional ICC reduced to 0.211, indicating that temperature and incubation time explained a substantial proportion of the observed variation in swimming behavior.

**Fig. 2:**
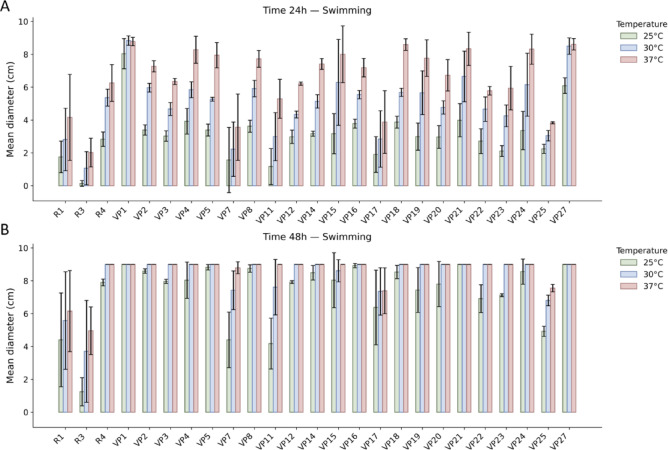
Swimming assays. Swimming of individual strains measured at 25 °C, 30 °C, and 37 °C after (**A**) 24 h and (**B**) 48 h of incubation (mean ± SD). Swimming was classified based on the diameter of the motility zone: none (0 cm), weak (0–4 cm), medium (4–8 cm), or strong (≥ 8 cm).

Swimming diameters varied across temperature and incubation time conditions (Fig. 2 and Supplementary Fig. S1). Mixed-effects Type III tests (Supplementary Table S3) revealed that diameters increased progressively with rising temperature (F = 76.65, *p* < 0.001) and prolonged incubation time (F = 368.31, *p* < 0.001). In addition, a significant time × temperature interaction (F = 36.92, *p* < 0.001) was observed.

Overall, pronounced variability in swimming behavior was observed across strains, with most strains displayed strong swimming motility (≥ 8 cm) after 48 h, particularly at 37 °C. Among all strains, VP1 exhibited the largest swimming expansion (9 cm).

### Motility (swarming)

Intraclass correlation analysis showed that 34.4% of the total variance in swarming behavior was attributable to strain identity (adjusted ICC = 0.344), which was lower than that observed for swimming motility (adjusted ICC = 0.554). After accounting for the fixed effects of temperature and incubation time, the conditional ICC decreased to 0.088, indicating that temperature and incubation time explained a substantial proportion of the overall variation in swarming behavior, leaving only a small proportion of the remaining variance attributable to strain identity.

**Fig. 3:**
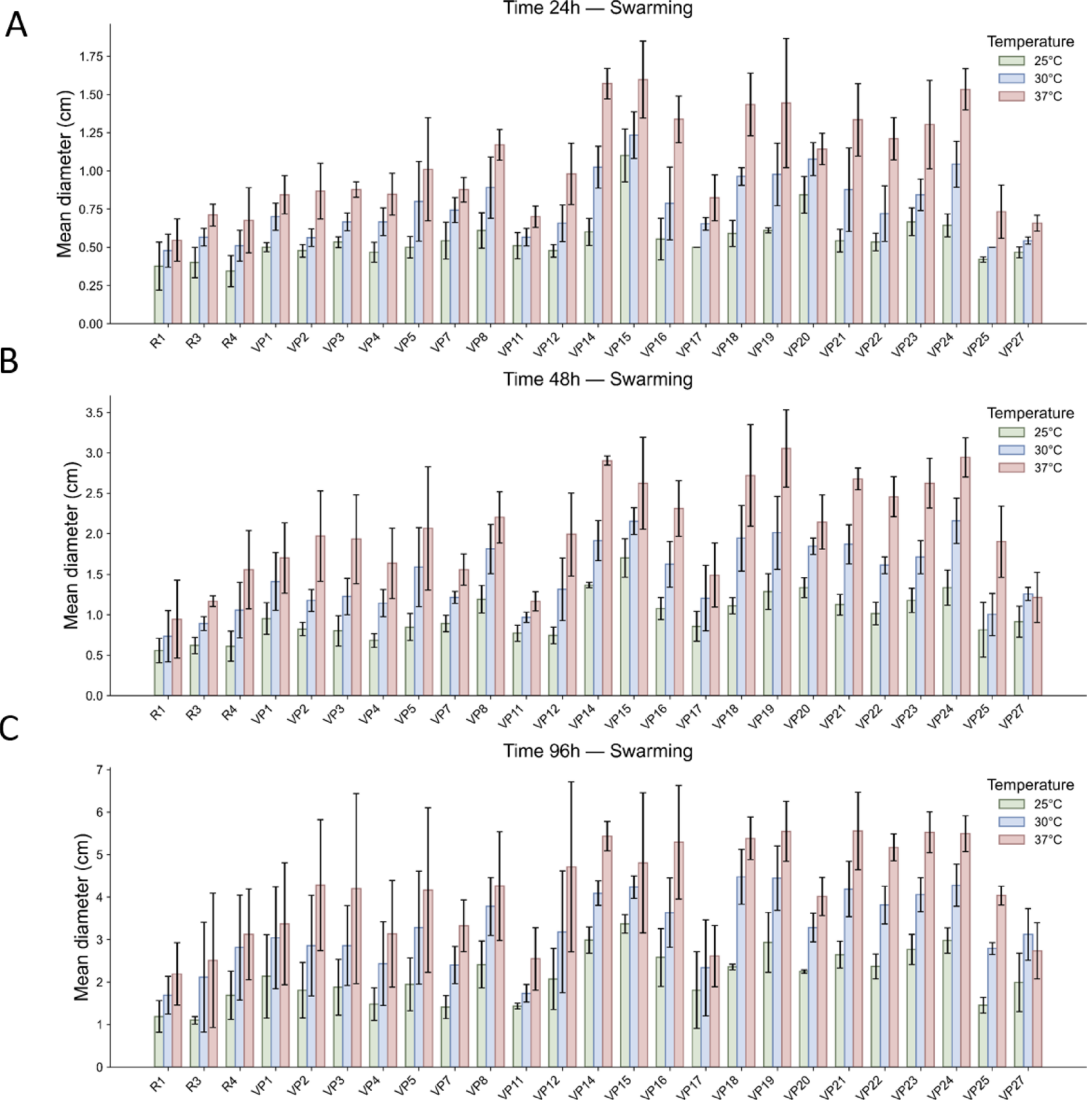
Swarming assays. Swarming motility of individual strains measured at 25 °C, 30 °C, and 37 °C after (**A**) 24 h, (**B**) 48 h, and (**C**) 96 h of incubation (mean ± SD).

Swarming behavior across temperatures and incubation times is illustrated in Fig. 3 and Supplementary Fig. S1. Swarming diameters increased significantly with incubation time (F = 331.90, *p* < 0.001) and rising temperature (F = 75.61, *p* < 0.001), with the greatest expansion observed at 37 °C and after 96 h, except for VP27 (Supplementary Table S4). Significant interactions were detected between time × temperature (F = 36.45, p < 0.001).

### Biofilm formation and CV assays

Intraclass correlation analysis demonstrated substantial strain-level heterogeneity, with 38.7% of the total variance attributable to differences in strain identity (adjusted ICC = 0.387). After accounting for temperature effects, strain-level variance remained high (conditional ICC = 0.319).

**Fig. 4:**
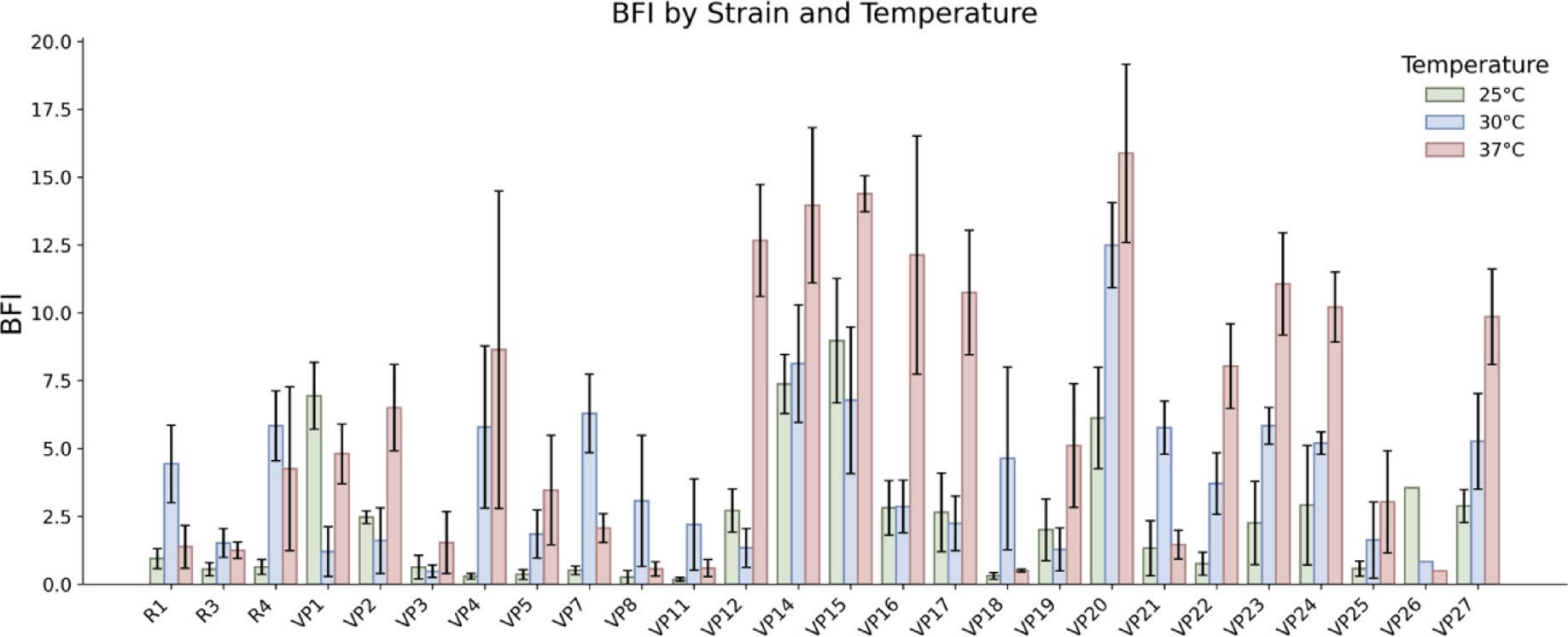
Strain-level biofilm formation index (BFI) of individual strains at 25 °C, 30 °C, and 37 °C (mean ± SD).

Temperature had a significant effect on biofilm formation index (BFI) (F = 7.89, *p* < 0.001). While most strains exhibited high biofilm formation at 37 °C, 8 strains reached their highest BFI at 30 °C (Fig. 4). Notably, 24 of strains exhibited high biofilm formation (BFI ≥ 1.10) at 30 °C. In contrast, biofilm formation at 25 °C was reduced in strains R1–R4, VP5, VP7, VP8, VP11, VP18, and VP25. Reduced biofilm formation was further observed in VP3 at 30 °C and in VP8 and VP11 at 37 °C.

### Association between motility traits (swimming, swarming) and biofilm formation

Associations among swimming, swarming, and BFI are shown in the Supplementary Table S5 and Supplementary Fig. S2. No significant correlations were observed between motility and BFI, whereas swimming and swarming were moderately correlated (*r* = 0.59, *p* < 0.001; R^2^ = 0.349).

### Distribution of motility-, biofilm- and virulence-associated genes across *V. parahaemolyticus* strains

Most genes were detected in all strains, as shown in Supplementary Fig. S3. In contrast, genes such as *pilA*, *calR*, *mshA*, and *mshD* were detected only in a subset of strains.

Strains VP23, VP11, VP12, VP22 and VP18 carried the highest gene richness across functional categories (adhesion, quorum sensing, biofilm, and T6SS2), lacking only *pilA* and *calR*. The strain VP2 lacked *oxyR, tnaA, mshA, mshD* and *pilA*. VP14 and VP15, lacked *tlh*. Among all strains, R1 was the only strain carrying *pilA*.

### Strain-level heterogeneity limits the predictive value of transcriptional profiles across *V. parahaemolyticus* strains

**Fig. 5:**
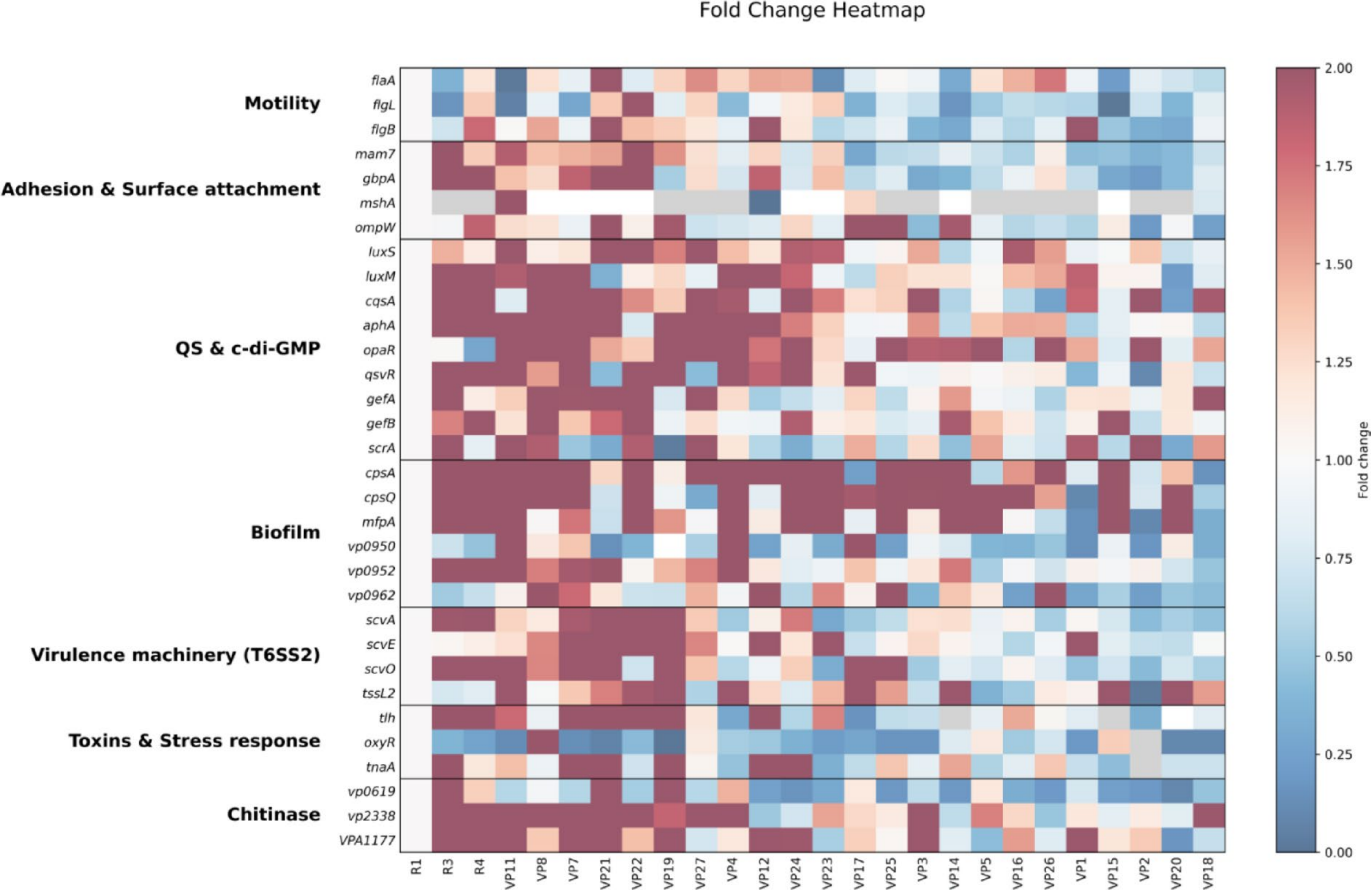
Fold-change heatmap of gene expression in *V. parahaemolyticus* strains. The heatmap depicts relative normalized expression (fold change, 0–2 scale) for motility-, adhesion-, QS/c-di-GMP-, biofilm-, T6SS2-, toxin/stress response-, and chitinase-associated genes. Fold changes were calculated relative to the reference strain R1 (RIMD 2,210,633), which served as the calibrator. Strains are ordered by decreasing number of genes with fold change > 1. Color intensity corresponds to fold-change magnitude from low (blue) to high (red). Grey cells indicate genes absent from the corresponding strain. White cells indicate genes that were present but showed no detectable expression under the experimental conditions, rather than representing missing data. Only quantified fold-change values are represented by the color scale.

**Fig. 6:**
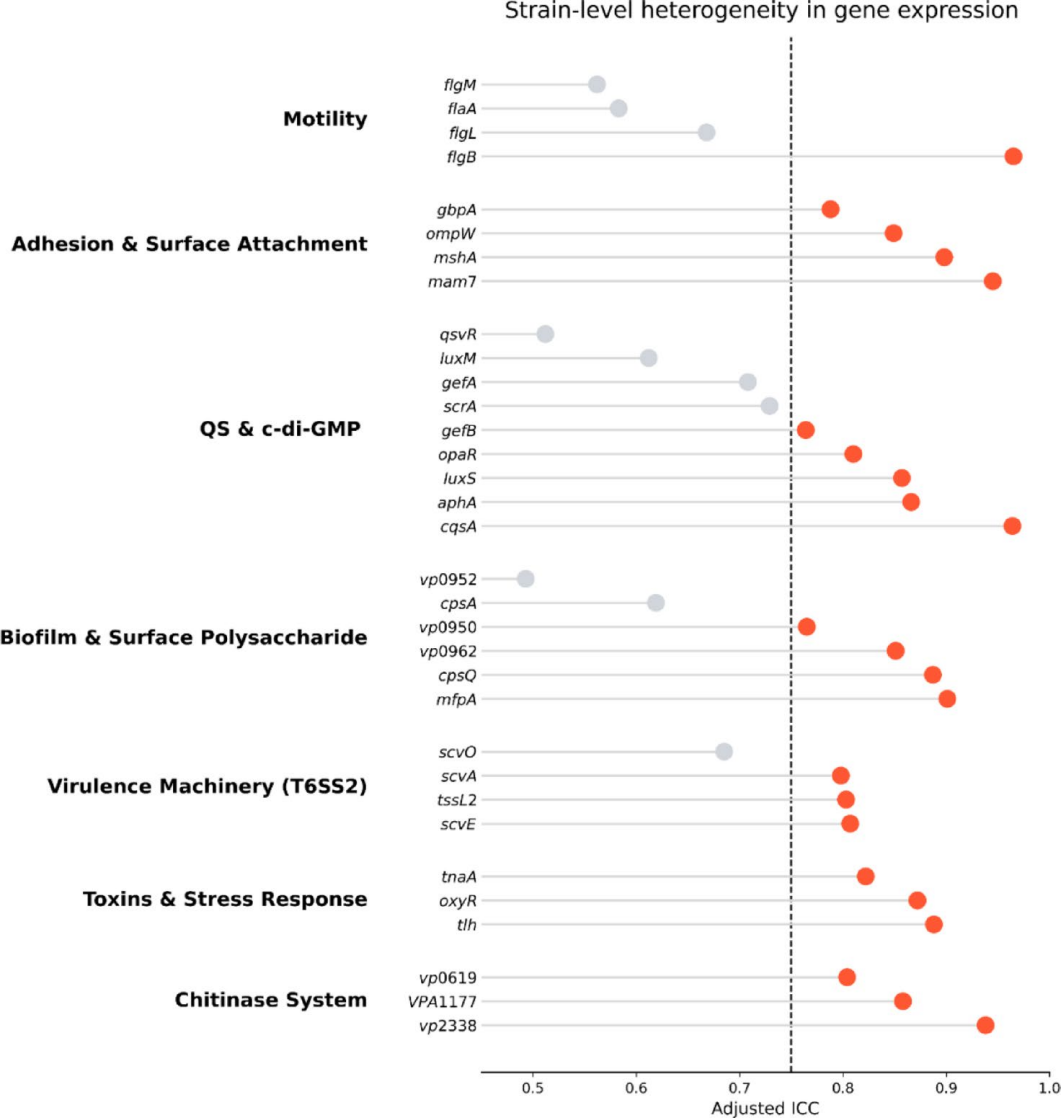
Intraclass correlation (ICC) analysis of gene expression. Gene-level ICC analysis reveals pronounced strain-level heterogeneity in transcriptional expression across strains. Higher ICC values indicate that a larger proportion of the total variance in gene expression is attributable to differences between strains rather than variation within strains, reflecting stronger strain-dependent transcriptional signatures.

Transcriptional expression patterns are shown in Fig. 5 and revealed substantial variability across individual strains. High biofilm-forming strains (VP7, VP14, VP15, VP18 and VP20) exhibited elevated expression of several EPS- and T6SS2-associated genes (Supplementary Fig. S4). ICC analysis (Fig. 6, Supplementary Table S6) showed that overall gene expression variability was predominantly driven by strain-level heterogeneity, with 69.7% of genes exhibiting adjusted ICC higher than 0.7.

**Fig. 7:**
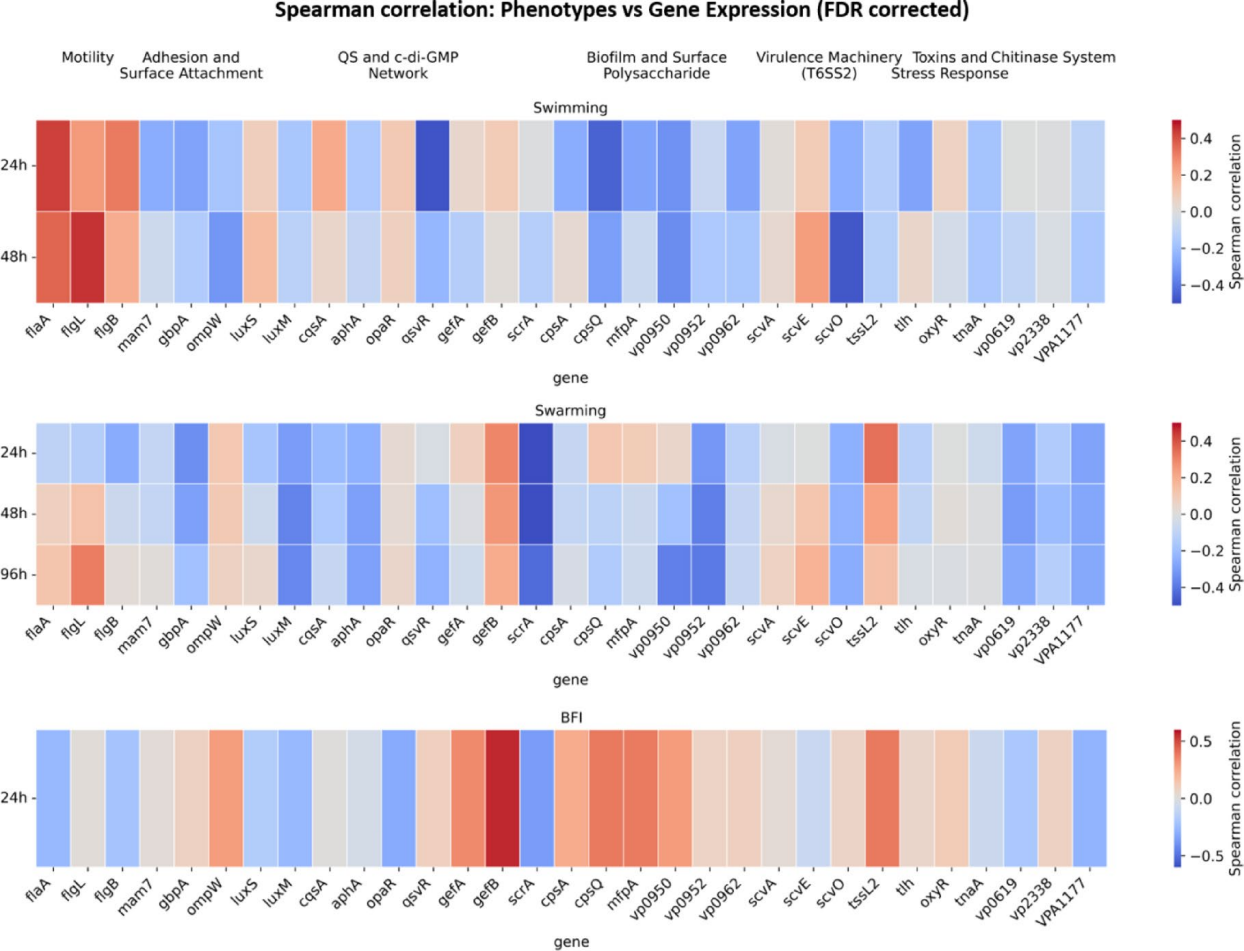
Spearman correlations between phenotypes and gene expression across *V. parahaemolyticus* strains. Heatmaps show Spearman correlation coefficients (*r*) between gene expression and swimming, swarming and BFI. All phenotypic assays were performed at 30 °C. Genes are grouped by functional categories as indicated. Color scale represents correlation strength and direction (red: positive; blue: negative).

**Fig. 8:**
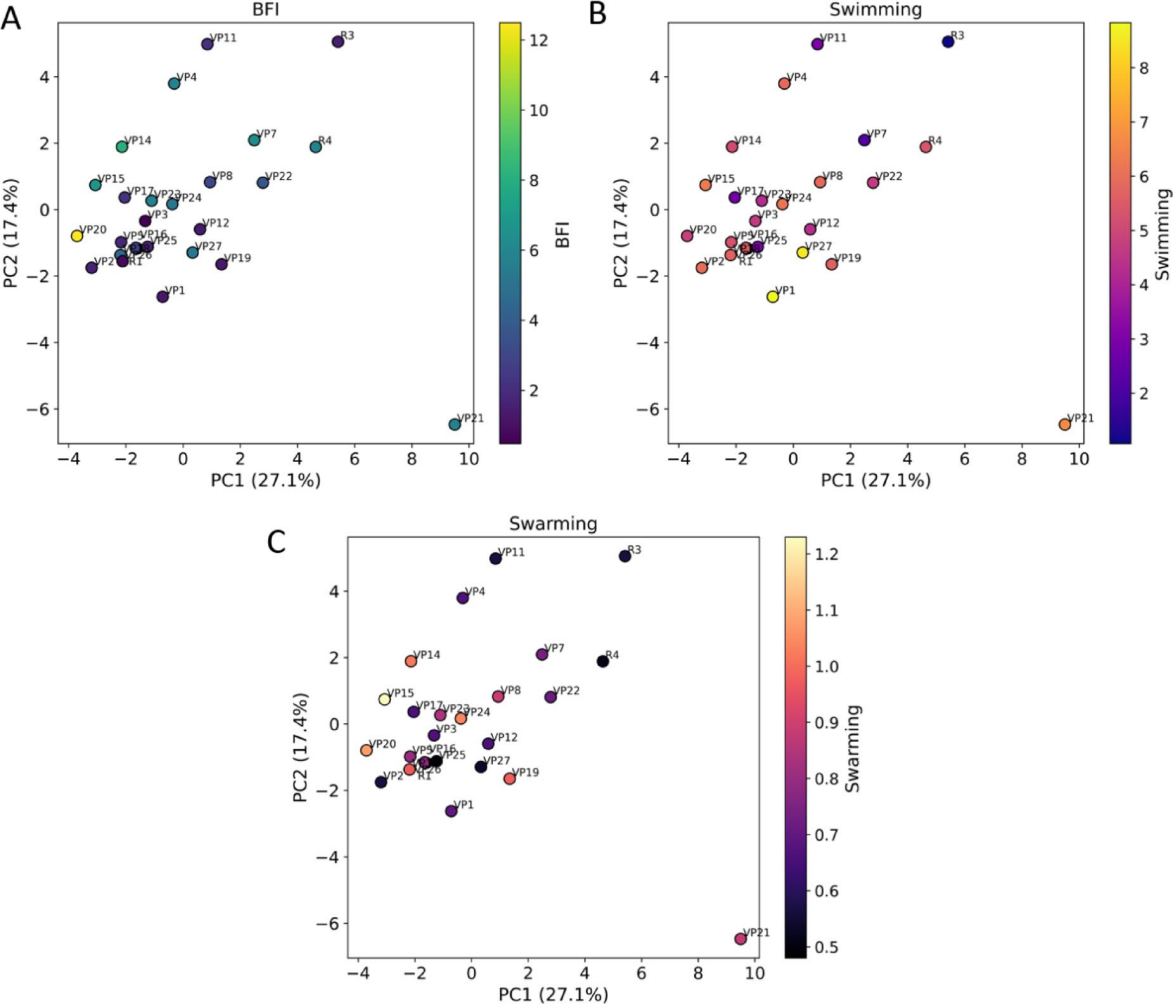
Principal component analysis (PCA) of planktonic transcriptional profiles colored according to phenotypic measurements obtained after 24 h at 30 °C. PCA was performed using the expression profiles of all quantified genes across all *Vibrio parahaemolyticus* strains. The same PCA coordinates are shown in all three panels, whereas point colors indicate the corresponding phenotypic values, allowing visual comparison between transcriptional similarity and phenotypic similarity across strains. Panels show (**A**) biofilm formation index (BFI), (**B**) swimming diameter, and (**C**) swarming diameter measured after 24 h at 30 °C. Principal component 1 (PC1) and principal component 2 (PC2) explain 27.1% and 17.4% of the total variance, respectively. Color gradients indicate the corresponding phenotypic values for each strain.

Spearman correlation analysis revealed no consistent associations between gene expression and motility- or biofilm phenotypes after Benjamini–Hochberg False Discovery Rate (FDR) correction (Fig. 7, Supplementary Tables S7–S9). Principal component analysis (PCA) analysis of all strains showed broad transcriptional variation across strains but did not reveal phenotype-associated clustering in the first two principal components. PC1 and PC2 together explained 44.5% of the total variance (Fig. 8).

Predictive modeling further supported these findings. To determine whether combinations of transcriptional markers could predict phenotypic variation across strains, least absolute shrinkage and selection operator (LASSO) regression with leave-one-out cross-validation (LOOCV) was performed. LASSO regression with LOOCV consistently showed poor predictive performance across all phenotypes and time points (Table 1). Negative R^2^ values were observed for swimming (24 h: − 0.41; 48 h: − 0.22), swarming (24 h: − 2.95; 48 h: − 7.83; 96 h: − 0.45), and biofilm formation (BFI: − 0.11), indicating that even combinations of genes failed to reliably predict phenotypic variation across strains.

**Table 1:** Predictive performance of LASSO regression models for phenotypic traits at 30 °C based on transcriptional profiles.

Phenotypes	LOOCV_R^2^	LOOCV_RMSE	Selected genes retained by LASSO
Swimming_24h	− 0.41	2.27	*aphA*, *flaA*, *flgB*, *oxyR*, *qsvR*
Swimming_48h	− 0.22	1.52	*cqsA*
Swarming_24h	− 2.95	0.37	No predictors retained
Swarming_48h	− 7.83	1.20	No predictors retained
Swarming_96h	− 0.45	1.00	No predictors retained
BFI_24h	− 0.11	2.10	No predictors retained

## Discussion

The study systematically demonstrates that strain-level heterogeneity dominates both phenotypic and transcriptional variation in *V. parahaemolyticus*, limiting the predictive value of baseline planktonic gene expression for surface-associated phenotypes across strains.

Substantial variability was observed across strains^39^ in swimming, swarming, and biofilm formation. ICC analysis showed evidence of strain-level heterogeneity in motility, suggesting that strain identity accounted for a larger proportion of variance in phenotypes, as previously observed for shellfish associated isolates^40^. Both swimming and swarming were associated with temperature and strongly influenced by incubation time, consistent with previous studies^31,41^.

Despite these phenotypic differences, transcriptional analyses revealed no consistent correlation between motility and gene expression. Under standardized planktonic growth conditions, variability in the expression of key motility-related genes, including the polar flagellar gene *flgL* and the quorum sensing regulator *opaR*, was observed across strains. However, Spearman correlation analysis revealed no significant associations between gene expression and swimming or swarming phenotypes after FDR correction. Given that motility in *V. parahaemolyticus* is governed by complex gene networks, increased transcription of individual genes may not directly translate into phenotypic differences^18,31,42,43^. Moreover, *opaR* expression measured under planktonic liquid cultures likely reflected quorum-sensing activity rather than surface-dependent repression of swarming, which requires surface sensing^44,45^.

ICC analysis also indicated that biofilm formation was strongly influenced by intrinsic differences among strains^46–48^. Even after accounting for temperature effects, considerable strain variability persisted, supported by the heterogeneous responses of individual strains. This aligns with the broad genomic and phenotypic diversity reported across *V. parahaemolyticus* populations observed in previous studies^40,49–52^.

Although biofilm formation generally increased with temperature, the temperature at which highest biofilm formation occurred varied among strains. At 25 °C, which approximates temperate coastal conditions, strains VP1, VP14, VP15, and VP20 exhibited enhanced biofilm formation under cooler, ocean-like environments^53^. At 30 °C, close to the optimal growth temperature of *V. parahaemolyticus*^54^, biofilm formation increased across most strains (Fig. 4). At 37 °C, most strains tended to maintain higher biofilm formation, whereas some strains showed comparatively lower levels (VP8, VP11, VP18, R1, and R3). This may indicate different physiological prioritization at elevated temperature across strains^53^. In comparison with other studies, strains examined in the present study exhibited higher BFI values under the experimental conditions, suggesting enhanced biofilm-forming capacity relative to strains isolated from shellfish^40^, food-poisoning cases^55^ and other marine hosts^56^.

Consistent with the findings for motility, expression of biofilm-associated genes did not reliably account for mature biofilm phenotypes. Under planktonic growth at 30 °C, some strains exhibited higher expression of several biofilm- and EPS-associated genes, including *gefB*, *cpsA*, *cpsQ*, *mfpA* and *vp0962*. However, Spearman correlation analysis revealed no significant associations after FDR correction, indicating that planktonic gene expression and biofilm formation capture distinct physiological states^52^, thus reflecting strain-specific regulatory tendencies rather than direct predictors of mature biofilm phenotypes^56^.

The absence of consistent relationships between motility and biofilm formation further supports the idea that these traits are not directly coupled under the conditions examined. Previous studies have reported inconsistent relationships between biofilm formation and motility, with both negative and positive associations described^17,56–59^. This apparent contradiction may be explained by the temporal sequence of surface colonization. Following surface attachment, swarming motility was downregulated while biofilm-associated pathways were activated^53^. This is in line with the model proposed by Trimble and McCarter^60^ that lateral flagellar expression and swarming motility can be suppressed by elevated c-di-GMP levels which in turn promote biofilm formation^21,29,61^.

In this context, swimming primarily facilitates dispersal and initial surface encounter^62^, and swarming represents an intermediate state preceding biofilm formation^60,63^. Consequently, a single transcriptional snapshot obtained during planktonic growth may not adequately represent phenotypes that emerge during later surface-associated stages, further limiting the predictive value of baseline transcriptional measurements.

Strain-level heterogeneity was also evident at the level of gene content. Several strains harbored a relatively conserved gene repertoire, while other strains exhibited greater gene-content diversity, likely reflecting adaptation to highly structured and competitive niches^64^.

Interestingly, strain VP14 and VP15 lacked the hemolysin gene *tlh* yet exhibited strong swarming motility and biofilm formation. This suggests that hemolysin genes are not required for these surface-associated phenotypes under the conditions tested, consistent with their established role as virulence factors rather than regulators of motility or biofilm formation^65^. Moreover, the frequent absence of *mshA*, *mshD*, and *calR* suggested that MSHA pili or *calR*-regulated pathways are not uniformly required for adhesion and biofilm formation across strains^40,57,66^.

Pronounced gene-content variability and the occurrence of strong surface-associated phenotypes in strains lacking canonical determinants indicate that the mere presence or absence of individual genes does not fully explain phenotypic variation. Moreover, even when genes are present, similar transcriptional levels may result in divergent functional outcomes across strains, thereby weakening the predictive relationship between planktonic gene expression and phenotype.

The term strain-level heterogeneity, as used in the present study, refers to the substantial phenotypic, transcriptional, and targeted gene-content variability observed among individual *V. parahaemolyticus* isolates. Such heterogeneity likely reflects underlying genomic diversity. Previous comparative genomic studies have shown that *V. parahaemolyticus* possesses an open pangenome with substantial accessory genome variation among strains, resulting in considerable differences in gene content beyond the conserved core genome^39^. Furthermore, comparative analyses have shown that strain-specific accessory gene repertoires may contribute to differences in biofilm-forming capacity and environmental adaptation^67^. In addition, genomic plasticity driven by mobile genetic elements, including genomic islands, prophages, integrative elements, and horizontal gene transfer, has been identified as an important source of strain diversification within *V. parahaemolyticus* populations^68^. Consequently, the phenotypic and transcriptional differences observed in the present study may reflect broader genomic variation that was not captured by the targeted gene panel used here.

For transcriptional profiles, ICC analysis demonstrated that gene expression variability was largely attributable to strain-level heterogeneity (Fig. 6). Many genes exhibited moderate to high ICC values, indicating that baseline transcriptional states were strongly shaped by strain identity. Thus, transcriptional variation was not randomly distributed across isolates but reflected consistent strain-specific expression patterns.

Interestingly, gene expression patterns varied across strains, but high biofilm-forming strains (VP7, VP14, VP15, VP18 and VP20) exhibited elevated expression of several EPS- and T6SS2-associated genes (Supplementary Fig. S4), consistent with a transcriptional tendency toward surface-associated traits^37,69^.

Importantly, no statistically significant correlation was observed between individual gene expression levels and phenotypes after FDR correction, indicating that baseline planktonic transcription alone provided limited predictive value for phenotypic variability across strains (Fig. 7). Although visual patterns in the heatmap suggested that motility-related genes tend to show correlation with swimming, and biofilm- and c-di-GMP-associated genes exhibit associations with BFI, these trends were not statistically supported after multiple testing correction.

PCA of all strains (Fig. 8) likewise did not reveal phenotype-associated clustering despite broad transcriptional variation across strains. Strains with similar swimming, swarming, or biofilm phenotypes were distributed throughout the PCA space rather than forming distinct clusters, indicating that similar phenotypic outcomes were associated with diverse baseline transcriptional profiles^70^.

This was supported by the poor predictive performance of LASSO models across all phenotypes and time points (Table 1). Unlike single-gene correlation analyses, LASSO regression evaluates whether combinations of transcriptional variables can jointly predict phenotypic variation. Despite incorporating multivariate transcriptional signatures, all models yielded negative cross-validated R^2^ values, indicating that baseline transcriptional profiles not only lack predictive power but fail to outperform mean-based predictions, suggesting a pronounced disconnect between transcriptional variation and phenotypic expression across strains^71^.

This lack of robust associations is consistent with the observation that c-di-GMP-related genes (*gefA*, *gefB*, *scrA*) were not differently expressed^30^, supporting previous findings that key regulatory pathways are primarily controlled at post-transcriptional and enzymatic levels^60,61,72^ rather than transcriptional regulation. While regulatory links between specific genes and biofilm formation have been demonstrated in single-strain models^13,72^, the present strain-resolved analysis suggests that these relationships did not generalize across diverse strains.

In addition, the discrepancy may also reflect differences between the laboratory biofilm assay used in this study and the natural ecological niche of *V. parahaemolyticus*. The biofilm assay was performed on abiotic polystyrene surfaces, whereas bacterium commonly colonizes chitin-containing surfaces in marine environments^73,74^. Chitin serves not only as a physical substrate but also as an environmental cue that activates regulatory pathways involved in surface sensing, biofilm development, and chitin utilization^40,75,76^. Accordingly, the chitin-associated genes examined in this study (*vp0619*, *vp2338*, and *VPA1177*) may be more relevant to chitin-dependent colonization than to biofilm formation on abiotic laboratory surfaces.

Taken together, transcriptional measurements under standardized planktonic conditions appear to reflect strain-specific regulatory states rather than generalizable predictors of multicellular surface-associated phenotypes across genetically diverse *V. parahaemolyticus* strains.

This study has several limitations. First, gene expression analyses were performed on planktonic cells at a single early exponential growth stage. While this approach was intentional and allowed standardized comparisons across strains, it did not capture the dynamic transcriptional changes occurring during later growth phases or surface-associated states. Second, while transcriptome-wide approaches such as RNA-seq would provide a more comprehensive view, the broad and targeted gene panel used here was selected to capture key regulatory and virulence-associated pathways, involved in motility, adhesion, quorum sensing, c-di-GMP signaling, and T6SS2 function, allowing for hypothesis-driven comparison across a large strain set. Third, whole-genome sequencing was not included in this study. Consequently, the genomic determinants underlying the observed phenotypic and transcriptional heterogeneity could not be assessed directly. Whole-genome sequencing would have enabled investigation of phylogenetic relationships, accessory genome variation, mobile genetic elements, and regulatory differences among isolates, thereby providing future insight into the biological basis of strain-level heterogeneity. Finally, transcriptional differences were not functionally validated at the protein or activity level. As a result, gene expression patterns should be interpreted as indicators of regulatory tendencies rather than direct predictors of phenotypic outcomes.

## Conclusion

In conclusion, this strain-resolved analysis demonstrates that baseline transcriptional states obtained under planktonic conditions do not reliably predict surface-associated phenotypes in *V. parahaemolyticus*. Instead, phenotypic variation is primarily driven by strain-level heterogeneity.

These findings highlight important limitations in extrapolating gene expression data from single-strain studies to surface-associated behaviors and challenge the common practice of inferring phenotypic traits from such data without considering strain-level heterogeneity. This underscores the need for strain-resolved and condition-specific approaches when linking transcriptional states to bacterial phenotypes.

## Methods

### *V. parahaemolyticus* strains and growth conditions

For phenotypic and genotypic analysis, a total of 26 *V. parahaemolyticus* isolates from diverse sources were included in this study. The strain collection comprised the type strain DSM 10027/ATCC 1780 and two additional reference strains DSM 11058/ATCC 43996 and DSM 101031/NCTC 10885 (German Collection of Microorganisms and Cell Cultures, Leibnitz-Institute, Braunschweig, Germany), as well as the pandemic strain RIMD 2210633 of serotype O3:K6 (Collection de l’Institut Pasteur). Furthermore, 23 isolates obtained from seafood matrices and seawater were included. These isolates were collected between 2023 and 2024 in a previous study^77^ or were part of the strain collection of the Institute of Food Safety and Food Hygiene, Freie Universität Berlin (Table 2).

**Table 2:** Twenty-six *V. parahaemolyticus* strains used in the study.

Matrix	Strain	Origin	Presence of *tdh* or *trh* gene
*Reference*			
	R1: RIMD 2210633/CIP 110006	clinical isolate Japan 1996	*tdh*
	R3: DSM 11058/ATCC 43996	cockles causing food poisoning	*tdh*
	R4: DSM 10027/ATCC 17802	shirasu food poisoning	*trh*
*Seafood matrices and seawater*			
Shrimp	VP1	India	
	VP2	Indonesia	
	VP3	Vietnam	
	VP4	Vietnam	
	VP5	India	
	VP7	Pacific	
	VP8	India	
	VP11	India	
	VP12	India	
	VP14	India	
	VP18	Unknown	*trh*
	VP19	Unknown	*trh*
	VP20	Baltic/North Sea	
	VP25	Unknown	*trh*
	VP26	unknown	*trh*
Squid	VP15	India	
Mussels	VP16	Mediterranean	
	VP17	Unknown	
Water	VP21	North Sea	
	VP22	sewage treatment plant	
	VP23	Baltic Sea	
	VP24	Kiel Canal	
Oysters	VP27: DSM 101031/NCTC 10885	England	

For the reference strains, “Origin” refers to the original isolation source. For the environmental isolates, it indicates the geographic origin where available. “Unknown” indicates that the geographic origin of the isolate was unavailable.

*tdh*, Thermostable direct hemolysin; *trh*, Thermostable direct hemolysin-related hemolysin.

For cultivation, *V. parahaemolyticus* strains were first streaked from cryopreserved stocks (Mast Diagnostica, Reinfeld, Germany) onto nutrient-rich lysogen broth (LB) agar plates (Sigma-Aldrich, St. Louis, MO, USA) and incubated for 24 h at 35 °C. A single colony from each plate was then inoculated into 5 mL tryptic soy broth (TSB) (Thermo Scientific, Waltham, MA, USA) containing 3% NaCl at 35 °C. The overnight cultures were obtained after incubation for 24 h.

### Swimming and swarming motility assays

Swimming and swarming were assessed using semi-solid and solid agar plates, respectively, as described previously with minor modifications^40^. Overnight cultures were prepared as described in “V. parahaemolyticus strains and growth conditions” section, and 1 µL of bacterial suspension was inoculated onto LB agar plates containing 3% NaCl, using agar concentrations of 0.3% for swimming and 0.5% for swarming assays. For swimming, plates were incubated in an upright position to minimize condensation disturbance. For swarming, plates were inverted and sealed in plastic bags during incubation to maintain adequate humidity. Plates were incubated at 25 °C, 30 °C, and 37 °C. The diameters of the motility zones were measured after 24 h and 48 h for swimming assays, and after 24 h, 48 h, and 96 h for swarming assays. Swimming and swarming ability was classified based on the diameter of the motility zone on the plate according to Zhang et al.^78^: none (0 cm), weak (0-4 cm), medium (4–8 cm), strong (≥ 8 cm).

### Biofilm formation and crystal violet assays

Biofilm formation was assessed using a crystal violet (CV) staining assay, according to previous established protocols^40^, with minor modifications. Overnight cultures obtained as described in “V. parahaemolyticus strains and growth conditions” section were standardized to an optical density at 595 nm (OD_595_) 0.15 ± 0.05 and each 200 µL of cell culture was transferred into flat-bottom 96-well microtiter plates. The edge effect was minimized by adding 200 µL of TSB containing 3% NaCl to the peripheral wells^79^. Plates were incubated statically at 25 °C, 30 °C, and 37 °C for 24 h.

After incubation, planktonic cell growth was measured by examining the OD_595_ on an EL800 Absorbance microplate reader (BioTek Instruments, Winooski, Vermont, USA). Bacterial cultures were discarded by inverting the plate and each well was gently washed three times using 200 µL of distilled water. Afterward, the plates were inverted on dry paper towels, gently tapped to remove excess liquid and air-dried overnight at room temperature.

Adhered cells were fixed with 200 µL of ethanol for 15 min at room temperature. The ethanol was removed, and the plates were allowed to dry for 1–1.5 h. Then, each well was stained with 200 µL of 0.1% CV for 30 min, washed three times by immersion in a large beaker filled with distilled water. Afterwards, the plates were inverted and tapped on dry paper towels to remove liquid and allowed to dry for 2 h at room temperature. CV stain was solubilized by adding 200 µL of 33% acetic acid to each well. The absorbance of the resulting solution was measured at 570 nm by Infinite® 200 PRO plate reader (Tecan Trading AG, Switzerland). Biofilm formation was quantified using BFI, calculated as follows:

$${\text{BFI }} = \frac{{\left( {OD_{570} - OD_{570con} } \right)}}{{\left( {OD_{595} - OD_{595con} } \right)}}$$

OD_570_ and OD_595_ represent the absorbance value of sample wells at 570 nm and 595 nm, respectively. OD_570con_ and OD_595con_ correspond to wells containing only TSB with 3% NaCl as blanks. Biofilm formation was classified according to Wang et al.^40^: strong (BFI ≥ 1.10), moderate (0.70 ≤ BFI ≤ 1.09), weak (0.35 ≤ BFI ≤ 0.69) and none (BFI < 0.35).

### Detection of motility-, biofilm- and virulence-associated genes

For the presence–absence analysis, real-time quantitative PCR (qPCR) was performed to screen for 35 motility-, biofilm- and virulence-associated genes (Table 3) using previously published oligonucleotide primer pairs listed in (Supplementary Table S1). The strain RIMD2210633 (R1), which contained all target genes in this study, served as the reference strain in gene detection and as a calibrator in gene expression analyses.

**Table 3:** Functional categorization of virulence- and biofilm-associated genes analyzed in this study.

Category	Genes
Motility	*flaA, flgL, flgB*
Adhesion and Surface Attachment	*mam7, gbpA, mshA, mshD, pilA, ompW*
QS and c-di-GMP Network	*luxS, luxM, cqsA, aphA, opaR, qsvR, gefA, gefB, scrA*
Biofilm and Surface Polysaccharide	*cpsA, cpsQ, mfpA, vp0950, vp0952, vp0962, calR*
Virulence Machinery (T6SS2)	*scvA, scvE, scvO, tssL2*
Toxins and Stress Response	*tlh, oxyR, tnaA*
Chitinase System	*vp0619, vp2338, VPA1177*

Bacterial DNA was extracted from pure cultures grown on LB agar supplemented with 3% NaCl. A single colony was suspended in 200 µL sterile distilled water. DNA was thermally lysed for 10 min at 95 °C, followed by centrifugation at 14,000 × g for 3 min. The resulting supernatant was used as the DNA template for qPCR analysis. Gene presence or absence was determined using a qPCR system (Bio-Rad, Hercules, CA, USA).

The PCR reactions (8 µL) contained 5.03 µL of 2 × SsoFast^TM^EvaGreen®Supermix (Bio-Rad), 0.12 µL of each of the primers (50 µM), 1.00 µL of the DNA template, and milli-Q water. The thermal cycling conditions consisted of an initial denaturation at 95 °C for 5 min, followed by 40 cycles of denaturation at 95 °C for 20 s, annealing/extension at 52 °C or 60 °C (depending on the primer-specific annealing temperature) for 20 s. After amplification, an additional denaturation step at 95 °C for 4 min was included to ensure complete strand separation prior to melting curve analysis. Melting curve analysis was subsequently conducted from 75 °C to 95 °C with 2 s increments to verify amplification specificity.

Amplification products were further confirmed by electrophoresis on 3% agarose gels (Biozym Biotech, Vienna, Austria) at 120 V for 50 min. Bands were visualized using a UV light E-BOX CX5 TS chamber (Vilber, Collégien, France). Primer efficiencies were in the range of 96% to 106%.

### Relative gene expression analysis under standardized planktonic growth conditions at 30 °C

#### Planktonic cell preparation

*V. parahaemolyticus* strains were first streaked from cryopreserved stocks onto LB agar plates and incubated for 24 h at 35 °C. A single colony of each strain was inoculated into 5 mL TSB containing 3% NaCl at 35 °C in a 180 rpm KS 4000 I control shaking incubator (IKA, Staufen, Germany). After 18 h incubation, bacterial culture was transferred into 300 mL Erlenmeyer flask containing 100 mL of TSB with 3% NaCl and standardized to an initial OD_600_ of 0.02 ± 0.01.

Cultures were grown at 30 °C with 180 rpm shaking until reaching the early exponential growth phase (OD_600_ 0.4 ± 0.05). The temperature of 30 °C was selected as an intermediate, controlled reference condition that enables standardized comparison across strains. Upon reaching the target value, 15 mL of culture was immediately placed on ice. After centrifugation at 4000 × g for 10 min at 4 °C, the supernatant was removed and 1 mL of RNAprotect Bacterial Reagent (Qiagen, Venlo, The Netherlands) was added. To verify the consistency of bacterial growth across cultures and to estimate the approximate cell numbers used for RNA stabilization (~ 10⁸ CFU), (OD_600_ 0.1 ± 0.05 and OD_600_ 0.5 ± 0.05), tenfold-dilution series in TSB were performed as shown in the (Supplementary Table S2). Then, 50 µL of dilutions were drop plated on LB agar plates and incubated for 24 h at 35 °C.

#### RNA extraction, cDNA synthesis, and RT-qPCR

Total RNA was extracted and purified from cells using QIAGEN RNA Mini Kit following manufacturer’s instructions. Total RNA was quantified using a NanoDrop ND 2000 spectrophotometer (Thermo Fisher Scientific). Each RNA sample was standardized to 1 µg and treated with DNase (Thermo Fisher Scientific) followed by complementary DNA (cDNA) synthesis through random hexamer primed reactions using a Maxima H first strand cDNA synthesis kit (Thermo Fisher Scientific). To verify the absence of genomic DNA contamination, no-reverse transcription (–RT) controls were included, in which reverse transcriptase was omitted and no amplification was observed in these controls. The resulting cDNA samples were subsequently diluted to a final concentration of 200 ng/µL for quantitative reverse transcription-polymerase chain reaction (RT-qPCR).

Melting curve analysis and primer efficiency validation for same genes as described in “Detection of motility-, biofilm- and virulence-associated genes” section were conducted to confirm amplification specificity. Primer efficiencies were similar to the previous detection analysis. The 16S rRNA and *recA* genes were initially evaluated as reference genes for normalization. Although *recA* has been shown to be one of the most stably expressed genes in *V. parahaemolyticus* strains cultured at different temperatures^80^, in a subset of strains consistently shifted Cq values (> 5 Cq) were observed for *recA* indicating that it was not suitable as a universal reference gene under the conditions applied in this study. The magnitude of these differences indicated variability in basal transcript abundance between strains rather than analytical variation. Therefore, normalization was performed using 16S rRNA only, as low Cq variability < 1 Cq was observed. Relative mRNA expression levels of target genes were calculated using the 2^−ΔΔCq^ method^81^, with data processing performed using CFX Maestro software (Bio-Rad).

### Statistical analysis

All experiments were performed with at least three independent biological replicates per strain, with at least three technical replicates. Data are presented as means ± standard deviation (SD) and were analyzed using IBM SPSS Statistics version 29.0 (IBM Corp., Chicago, IL, USA).

Model assumptions for linear mixed-effects models were evaluated by visual inspection of residual distributions and Q–Q plots to assess normality and homoscedasticity of residuals. All statistical tests were two-tailed.

Phenotypic and transcriptional data were analyzed using linear mixed-effects models, with strain identity included as a random effect and temperature and time included as fixed effects where applicable. Intraclass correlation coefficients (ICC) were calculated from the mixed-effects models to estimate the proportion of total variance attributable to strain identity. Both adjusted and conditional ICC values were obtained from the model output. PCA was performed on gene expression of all quantified genes across strains. Correlations between gene expression and phenotypes were evaluated using Spearman’s correlation analysis with *p*-values adjusted by the FDR method. Gene expression analyses were performed only for genes that were present and successfully quantified across the strain collection. Genes absent from the majority of isolates (*mshD*, *pilA*, and *calR*) were excluded from expression analyses.

To assess predictive performance, LASSO regression with LOOCV was applied using gene expression as predictors and phenotypic traits as response variables. Model performance was evaluated using cross-validated R^2^ and root mean squared error (RMSE). For each phenotype and time points, all transcriptional variables were initially included as candidate predictors. LASSO regression was then used to perform automated variable selection.

Data processing, statistical analysis, and visualization were additionally performed in R (v4.5.1) and Python (v3.11).

## Supplementary Information

Below is the link to the electronic supplementary material.

Supplementary Material 1

## Data Availability

All data supporting the findings of this study are included within the article and its Supplementary Materials. Raw data will be available on request.
