# Supplementary material for: Strain-level heterogeneity in Vibrio parahaemolyticus limits the predictive value of baseline planktonic gene expression for surface-associated phenotypes
Source: Sci Rep. 2026 Jul 27;16:23322. doi: 10.1038/s41598-026-64000-1 (PMC13408953; doi:10.1038/s41598-026-64000-1)
Supplement: Supplementary file 1 — Supplementary Material 1 [file 41598_2026_64000_MOESM1_ESM.docx]

**Supplementary Material**

**Table S1.** Primers used in the study.

| **Primer** | **Category** | **Function** | **Sequence (5‘to 3‘) F/R** | **Gene ID** | **Reference Sequence (GenBank accession)** | **Amplicon size(bp)** | **Reference** |
| --- | --- | --- | --- | --- | --- | --- | --- |
| *flaA* | Motility | Polar flagella | CGGACTAAACCGTATCGCTGAAA  /GGCTGCCCATAGAAAGCATTACA | Not found | GQ433373.1 | 128 | [82] |
| *flgB* | Motility | Polar flagella | GTACATCCAGAGGCACTCAAT  /AACTTAGGTCTTTCGCCAAGTA | 1190952 | NC_004605.1 | 103 | [22] |
| *flgL* | Motility | Polar flagella | CGTCAGCGTCCACCACTT /GCGGCTCTGACTTACTGCTA | 1190962 | CP066246.1 | 141 | [82] |
| *mam7* | Adhesion | Multivalent Adhesion Molecule 7; mediating host binding and initiation of infection. | TTTGGCGTCAGCAGCAATTC /CGCCAGGAACGATAGTGAGG | 1189118 | NC_004603.1 | 149 | [54] |
| *gbpA* | Adhesion | Chitin/GlcNAc-binding adhesin mediating environmental and intestinal attachment. | CCTCACTCTTACCGCAGAGG /CAAGGTTGCCATCGGTATCT | 1192294 | NC_004605.1 | 191 | [54] |
| *mshA* | Adhesion | Mannose-sensitive haemagglutinin (MSHA) crucial for surface adhesion and early biofilm attachment. | GCTTAGCCGTACTCGTTGCT /TGTAGTTGGCGGAGCATACA | 1191436 | NC_004605.1 | 160 | [54,83] |
| *mshD* | Adhesion | MSHA pilus-associated protein contributing to adhesion and biofilm formation. | GCG TGG TTT TGA TGA GAA CAG /TCA GTG TCA GAG GTT GAG TG | 1190240 | NC_004603.1 | 368 | [84] |
| *pilA* | Adhesion | Type IV pili, adhesion, biofilm formation | GGCTGAGCTGCATTACCAAG /TTGCAGCGTCTCTTGTGAAC | 1190038 | NC_004603.1 | 194 | [54,83] |
| *ompW* | Surface Attachment | Outer membrane protein involved in surface interaction and environmental adaptation | TCG TGT CAC CAA GTG TTT TCG /CGT GGC TGA ATG GTG TTG C | 1190775 | NC_004605.1 | NA* | [84] |
| *luxS* | QS | Autoinducer binding domain-containing protein;QS regulator | GGATTTTGTTCTGGCTTTCCACTT /GGGATGTCGCACTGGTTTTTAC | 1190052 | CP066246.1 | 119 | [82] |
| *luxM* | QS | HAI-1 (AI-1) autoinducer synthase of the Vibrio QS system. | TGCCCTTGTTGTCACTTTCT  /CGTTGGTTCCAGTCTTGGATTA | Not found | Not found | NA* | [21,85] |
| *aphA* | QS | Acid phosphatase AphA; Low-cell-density master regulator promoting adhesion and early biofilm genes. | ACACCCAACCGTTCGTGATG /GTTGAAGGCGTTGCGTAGTAAG | 1190312 | CP066246.1 | 162 | [82] |
| *opaR* | QS | Transcriptional regulator OpaR; High-cell-density master regulator repressing motility and modulating virulence/biofilm. | TGTCTACCAACCGCACTAACC /TTGAAGCGGTTTGCTTGTACG | 1190031 | NC_004603.1 | 154 | [54,67] |
| *qsvR (VPA0606)* | QS | AraC-type transcriptional regulator QsvR; QS regulator | TAC ACC GCC ACC CAT AAC G /AGC CAT TCT CGC CAG GTA TG | 1191295 | NC_004605.1 | 157 | [86] |
| *cqsA* | QS | QS regulator, exhibits swarming defects in the absence of CqsA, LuxMvp | ACTTCCACACTCAAGAGCAATA  /GTTCAAGCGAGCCAAAGAAC | 1191400 | NC_004605.1 | 94 | [21] |
| *gefA (VPA0202)* | c-di-GMP | GGDEF domain -cyclic di-GM, increases c-di-GMP and promotes EPS/biofilm formation. | CGAAGAAGTGATGGTGGTG /CTCGCATTGGTGAGTTGACG | 1190890 | NC_004605.1 | NA* | [59,87] |
| *gefB (VPA1478)* | c-di-GMP | GGDEF domain -cyclic di-GM enhancing EPS production and repressing motility. | CCTGACTTTCCCATTTACT /CTTGCTTTGACCGACAGTA | 1192174 | NC_004605.1 | 319 | [59,87] |
| *scrA* | c-di-GMP | Part of the ScrABC (VPA1513-11) surface-sensing system regulating GGDEF/EAL activity and motility–biofilm switching. | CTAATCGCAGCCGACGTAAT  /GAGCTTGTGCTACACCTGAA | 1192209 | NC_004605.1 | 93 | [60,88] |
| *vp0950* | Biofilm | Biofilm-associated surface proteins linked to persistence and colonization | GCCAAACTTCTCAAACAACA /ATGAAACGCAATTTACCATC | 1188454 | NC_004603.1 | 304 | [89] |
| *vp0952* | Biofilm | Biofilm-associated surface proteins linked to persistence and colonization | TATGATGGTGTTTGGTGC  /TGTTTTTCTGAGCGTTTC | 11884  56 | CP064041.1 | 276 | [8,82] |
| *vp0962* | Biofilm | Biofilm-associated surface proteins linked to persistence and colonization | GACCAAGACCCAGTGAGA  /GGTAAAGCCAGCAAAGTT | 1188466 | CP064041.1 | 358 | [8,82] |
| *cpsA* | Biofilm | Capsular polysaccharide biosynthesis gene involved in EPS production. | GAGAGCGGCAACCTATATCG /GCGGTCAAACAAAGGGTAAAC | 1192099 | NC_004605.1 | 141 | [33,90,91] |
| *cpsQ (VPA1446)* | Biofilm | LuxR-family transcriptional activator of CPS and biofilm-related operons. | GCC TGA AATCCTAATGCTC /AGTGTC AGA AGG TGTATC AAC | 1192142 | NC_004605.1 | 181 | [12,36] |
| *mfpA (VPA1443)* | Biofilm | Membrane fusion protein; matrix-associated membrane fusion protein of the cpsQ–mfpABC operon. | GCG GGC AATGATCGTCTA AC /TCACCTGAACCTGCG ACA AG | 1192139 | NC_004605.1 | 170 | [12,36] |
| *calR* | Biofilm | LysR-type transcriptional regulator; T3SS/T6SS, biofilm formation. | ATG TAA AAAGAA AACCGTACA /AAC ACAGCAGAATGACCG TG | 1187817 | NC_004605.1 | 157 | [12,36] |
| *scvA* | Virulence Machinery (T6SS2) | EPS biosynthesis; T6SS2 structural components. | ACCCAAGACAAACTAGCGATTG /CGTGCTGGAGGTTGATTTCG | 1188983 | NC_004603.1 | 130 | [33,92] |
| *scvE* | Virulence Machinery (T6SS2) | EPS biosynthesis; T6SS2 structural components. | GACAGGTCGTGATGCCATTC /GGCGATGATGACCGAAGTG | 1188987 | NC_004603.1 | 141 | [19,33,92] |
| *scvO* | Virulence Machinery (T6SS2) | EPS biosynthesis; T6SS2 structural components. | GTTCATTGCTCTTGCCATCA /CGAGGGCCAATCATAGACAT | 1188997 | NC_004603.1 | 276 | [19] |
| *tssL2 (VPA1040)* | Virulence Machinery (T6SS2) | Core membrane-anchoring protein of T6SS2; deletion affects motility and biofilm phenotypes. | CTTCCGCACTGCTTGTTTGT /AACTGCTGGGGTTGGTTGAA | 1191735 | NC_004605.1 | 110 | [12] |
| *tlh* | Toxins & Stress Response | Thermolabile hemolysin; species marker and cytotoxic virulence factor. | AAA GCG GAT TAT GCA GAA GCA CTG /GCT ACT TTC TAG CAT TTT CTC TGC | 1190914 | NC_004605.1 | 450 | [93] |
| *oxyR* | Toxins & Stress Response | Oxidative stress response regulator controlling ROS-defense and biofilm/motility modulation. | TCG TCA GCT AGA GGA AGG /TGG TCG CGT AAG CAA TGC | 1190302 | NC_004603.1 | 210 | [10] |
| *tnaA* | Toxins & Stress Response | Tryptophanase; produce indole inhibit biofilm formation | TGTACGAAATTGCCACCAAA /TCAGCGTAACCTTCTTCACG | 1190879 | NC_004605.1 | 103 | [84] |
| *vp0619* | Chitinase | ChiB-like chitinase enabling growth on chitin and natural competence. | GTGATGCATCTGTATTGCTG  /CCAAGTCCGGTCAATTACATG | 1188094 | NC_004603.1 | 134 | [34] |
| *vp2338* | Chitinase | Chitinase facilitating environmental survival on crustacean surfaces. | GATTGCGACAGACAATGAAG  /GTGAGAGTAATGCTAGGTGG | 1189851 | NC_004603.1 | 96 | [34] |
| *VPA1177* | Chitinase | Secreted chitinase involved in chitin degradation and nutrient acquisition | CTCAGTTAACGTGCCATACAAAG  /CATTGTCCAACCACCAAAAGAAG | 1191873 | NC_004605.1 | 103 | [34] |
| 16S rRNA | Housekeeping gene (reference) | 16S ribosomal RNA; conserved bacterial rRNA gene used as an internal reference for normalization. | ACGGCCTGGGGAGTACGGTC  /TTGCGCTCGTTGCGGGACTT | NA* | NC_004605.1 | NA* | This study |
| *recA* | Housekeeping gene (reference) | Recombinase A; ATP-dependent DNA strand exchange protein; commonly used as a housekeeping reference gene for normalization. | GAAACCATTTCAACGGGTTC  /GTGCAGCAGCGATAAGCTC | 1190074 | NC_004603.1 | 139 | This study |

**NA, not available. The predicted in silico amplicon sizes were approximately 120 bp for luxM, 210 bp for ompW, and 100 bp for gefA.*

**Table S2.** Total viable cell counts (log₁₀ CFU/mL) of *V. parahaemolyticus* strains at two initial inoculation densities

| **Strain** | **OD_600_ 0.1 (Mean ± SD, log_10_ CFU/mL)** | **OD_600_ 0.5 (Mean ± SD, log_10_ CFU/mL)** |
| --- | --- | --- |
| R1 | 8.0 ± 0.1 | 8.4 ± 0.1 |
| R3 | 7.5 ± 0.2 | 8.4 ± 0.1 |
| R4 | 7.9 ± 0.2 | 8.2 ± 0.0 |
| VP1 | 7.8 ± 0.2 | 8.1 ± 0.5 |
| VP2 | NA*** | NA*** |
| VP3 | 7.5 ± 0.2 | 8.4 ± 0.2 |
| VP4 | 7.5 ± 0.3 | 8.2 ± 0.0 |
| VP5 | 7.3 ± 0.0 | 8.4 ± 0.1 |
| VP7 | 8.2 ± 0.0 | 8.8 ± 0.1 |
| VP8 | 5.8 ± 3.0 | 8.4 ± 0.5 |
| VP11 | 7.8 ± 0.2 | 8.4 ± 0.0 |
| VP12 | 8.2 ± 0.2 | 8.6 ± 0.0 |
| VP14 | 8.3 ± 0.3 | 8.7 ± 0.0 |
| VP15 | 7.7 ± 0.1 | 8.4 ± 0.0 |
| VP16 | 7.9 ± 0.0 | 8.5 ± 0.0 |
| VP17 | 7.3 ± 0.0 | 8.4 ± 0.0 |
| VP18 | 7.5 ± 0.3 | 8.0 ± 0.0 |
| VP19 | 7.8 ± 0.2 | 8.4 ± 0.2 |
| VP20 | 7.1 ± 0.1 | 7.7 ± 0.5 |
| VP21 | 7.8 ± 0.3 | 8.5 ± 0.1 |
| VP22 | 7.5 ± 0.2 | 8.5 ± 0.1 |
| VP23 | 7.8 ± 0.2 | 8.3 ± 0.2 |
| VP24 | 7.7 ± 0.6 | 8.3 ± 0.1 |
| VP25 | 7.8 ± 0.3 | 8.6 ± 0.2 |
| VP26 | 7.5 ± 0.3 | 8.2 ± 0.2 |
| VP27 | 7.5 ± 0.0 | 8.2 ± 0.2 |

**NA, not available. Mean ± SD of* *log₁₀ CFU/mL determined by tenfold serial dilution in TSB.*

**Table S3**. Type III tests of fixed effects for swimming

| **Source** | **F** | ***p*-value** |
| --- | --- | --- |
| Time | 368.31 | <0.001 |
| Temperature | 76.65 | <0.001 |
| Time × Temperature | 36.92 | <0.001 |

**Table S4**. Type III tests of fixed effects for swarming

| **Source** | **F** | ***p*-value** |
| --- | --- | --- |
| Time | 331.90 | <0.001 |
| Temperature | 75.61 | <0.001 |
| Time × Temperature | 36.45 | <0.001 |

**Table S5**. Pearson correlation coefficients among motility traits and biofilm formation at 24 h across all temperatures. (*p*<0.05)

| **Variable pair** | ***r*** | ***p*-value** |
| --- | --- | --- |
| Swimming – Swarming | 0.59 | <0.001 |
| Swimming – BFI | 0.00 | 0.997 |
| Swarming – BFI | 0.09 | 0.166 |

**Table S6.** Adjusted intraclass correlation coefficients (ICC) for gene expression across strains.

| **Gene** | **Intraclass Correlation Coefficients Adjusted** |
| --- | --- |
| *aphA* | 0.866 |
| *cpsA* | 0.619 |
| *cpsQ* | 0.887 |
| *cqsA* | 0.964 |
| *flaA* | 0.583 |
| *flgB* | 0.965 |
| *flgL* | 0.668 |
| *flgM* | 0.562 |
| *gbpA* | 0.788 |
| *gefA* | 0.708 |
| *gefB* | 0.764 |
| *luxM* | 0.612 |
| *luxS* | 0.857 |
| *mam7* | 0.945 |
| *mfpA* | 0.901 |
| *mshA* | 0.898 |
| *ompW* | 0.849 |
| *opaR* | 0.810 |
| *oxyR* | 0.872 |
| *qsvR* | 0.512 |
| *scrA* | 0.729 |
| *scvA* | 0.798 |
| *scvE* | 0.807 |
| *scvO* | 0.685 |
| *tlh* | 0.888 |
| *tnaA* | 0.822 |
| *tssL2* | 0.803 |
| *vp0619* | 0.804 |
| *vp0950* | 0.765 |
| *vp0952* | 0.493 |
| *vp0962* | 0.851 |
| *vp2338* | 0.938 |
| *VPA1177* | 0.858 |

**Table S7.** Spearman correlation between gene expression levels and swimming at 30 °C across all strains.

| **Gene** | ***r*_24h** | ***p*_24h** | ***r*_48h** | ***p*_48h** | ***p*_24_FDR** | ***p*_48_FDR** |
| --- | --- | --- | --- | --- | --- | --- |
| *VPA1177* | -0.11 | 0.60 | -0.16 | 0.45 | 0.92 | 0.91 |
| *aphA* | -0.16 | 0.44 | -0.09 | 0.68 | 0.91 | 0.95 |
| *cpsA* | -0.26 | 0.22 | 0.03 | 0.88 | 0.76 | 0.97 |
| *cpsQ* | -.447* | 0.03 | -0.30 | 0.14 | 0.39 | 0.75 |
| *cqsA* | 0.20 | 0.33 | 0.05 | 0.82 | 0.91 | 0.97 |
| *flaA* | 0.431* | 0.03 | 0.37 | 0.07 | 0.39 | 0.75 |
| *flgB* | 0.32 | 0.13 | 0.20 | 0.36 | 0.75 | 0.91 |
| *flgL* | 0.25 | 0.23 | 0.450* | 0.02 | 0.76 | 0.39 |
| *gefA* | 0.04 | 0.84 | -0.13 | 0.52 | 0.97 | 0.92 |
| *gefB* | 0.10 | 0.64 | 0.01 | 0.95 | 0.92 | 0.99 |
| *gbpA* | -0.29 | 0.17 | -0.14 | 0.49 | 0.75 | 0.92 |
| *luxM* | -0.17 | 0.44 | -0.11 | 0.59 | 0.91 | 0.92 |
| *luxS* | 0.08 | 0.70 | 0.15 | 0.48 | 0.95 | 0.92 |
| *mam7* | -0.26 | 0.21 | -0.06 | 0.77 | 0.76 | 0.95 |
| *ompW* | -0.17 | 0.41 | -0.32 | 0.12 | 0.91 | 0.75 |
| *opaR* | 0.11 | 0.61 | 0.07 | 0.72 | 0.92 | 0.95 |
| *mfpA* | -0.28 | 0.17 | -0.07 | 0.74 | 0.75 | 0.95 |
| *qsvR* | -0.482* | 0.01 | -0.22 | 0.28 | 0.39 | 0.83 |
| *scrA* | -0.01 | 0.97 | -0.13 | 0.53 | 0.99 | 0.92 |
| *oxyR* | 0.07 | 0.75 | -0.04 | 0.85 | 0.95 | 0.97 |
| *scvE* | 0.10 | 0.64 | 0.24 | 0.25 | 0.92 | 0.78 |
| *tnaA* | -0.18 | 0.38 | -0.16 | 0.44 | 0.91 | 0.91 |
| *tssL2* | -0.13 | 0.54 | -0.13 | 0.55 | 0.92 | 0.92 |
| *vp0619* | 0.00 | 0.99 | -0.10 | 0.63 | 0.99 | 0.92 |
| *vp0950* | -0.33 | 0.11 | -0.34 | 0.10 | 0.75 | 0.75 |
| *vp0962* | -0.28 | 0.17 | -0.16 | 0.43 | 0.75 | 0.91 |
| *vp0952* | -0.07 | 0.73 | -0.16 | 0.46 | 0.95 | 0.91 |
| *vp2338* | 0.00 | 0.98 | -0.02 | 0.94 | 0.99 | 0.99 |
| *tlh* | -0.28 | 0.19 | 0.05 | 0.82 | 0.76 | 0.97 |
| *scvO* | -0.25 | 0.23 | -.476* | 0.02 | 0.76 | 0.39 |
| *scvA* | 0.01 | 0.94 | 0.04 | 0.86 | 0.99 | 0.97 |

*Means significant, *p*<0.05

**Table S8.** Spearman correlation between gene expression levels and swarming at 30 °C across all strains.

| **Gene** | ***r*_24** | ***p*_24** | ***r*_48** | ***p*_48** | ***r*_96** | ***p*_96** | ***p*_24_FDR** | ***p*_48_FDR** | ***p*_96_FDR** |
| --- | --- | --- | --- | --- | --- | --- | --- | --- | --- |
| *VPA1177* | -0.29 | 0.17 | -0.28 | 0.19 | -0.27 | 0.21 | 0.78 | 0.78 | 0.78 |
| *aphA* | -0.24 | 0.24 | -0.29 | 0.15 | -0.29 | 0.16 | 0.78 | 0.78 | 0.78 |
| *cpsA* | -0.08 | 0.69 | -0.09 | 0.67 | -0.03 | 0.88 | 1.00 | 1.00 | 1.00 |
| *cqsA* | -0.21 | 0.31 | -0.16 | 0.45 | -0.08 | 0.69 | 0.89 | 1.00 | 1.00 |
| *cpsQ* | 0.11 | 0.59 | -0.11 | 0.60 | -0.15 | 0.48 | 1.00 | 1.00 | 1.00 |
| *flgB* | -0.26 | 0.22 | -0.07 | 0.76 | 0.02 | 0.91 | 0.78 | 1.00 | 1.00 |
| *gbpA* | -0.34 | 0.09 | -0.30 | 0.15 | -0.19 | 0.36 | 0.78 | 0.78 | 0.93 |
| *gefA* | 0.08 | 0.72 | -0.01 | 0.96 | -0.05 | 0.82 | 1.00 | 1.00 | 1.00 |
| *gefB* | 0.29 | 0.15 | 0.26 | 0.22 | 0.20 | 0.33 | 0.78 | 0.78 | 0.91 |
| *luxM* | -0.30 | 0.15 | -0.37 | 0.08 | -0.36 | 0.09 | 0.78 | 0.78 | 0.78 |
| *mam7* | -0.09 | 0.66 | -0.09 | 0.67 | 0.01 | 0.97 | 1.00 | 1.00 | 1.00 |
| *mfpA* | 0.09 | 0.65 | -0.06 | 0.76 | -0.06 | 0.77 | 1.00 | 1.00 | 1.00 |
| *ompW* | 0.11 | 0.59 | 0.11 | 0.61 | 0.07 | 0.74 | 1.00 | 1.00 | 1.00 |
| *oxyR* | 0.00 | 0.99 | 0.02 | 0.94 | -0.01 | 0.95 | 1.00 | 1.00 | 1.00 |
| *qsvR* | -0.02 | 0.92 | -0.20 | 0.35 | -0.24 | 0.24 | 1.00 | 0.93 | 0.78 |
| *opaR* | 0.03 | 0.88 | 0.03 | 0.89 | 0.05 | 0.82 | 1.00 | 1.00 | 1.00 |
| *scrA* | -.503* | 0.01 | -.495* | 0.01 | -.428* | 0.03 | 0.55 | 0.55 | 0.78 |
| *scvA* | -0.02 | 0.91 | 0.04 | 0.84 | 0.07 | 0.75 | 1.00 | 1.00 | 1.00 |
| *scvE* | 0.00 | 1.00 | 0.12 | 0.57 | 0.18 | 0.39 | 1.00 | 1.00 | 0.95 |
| *scvO* | -0.25 | 0.24 | -0.24 | 0.25 | -0.26 | 0.21 | 0.78 | 0.78 | 0.78 |
| *tlh* | -0.12 | 0.59 | -0.09 | 0.67 | -0.02 | 0.93 | 1.00 | 1.00 | 1.00 |
| *tnaA* | -0.06 | 0.78 | -0.01 | 0.98 | -0.01 | 0.98 | 1.00 | 1.00 | 1.00 |
| *tssL2* | 0.34 | 0.10 | 0.23 | 0.27 | 0.14 | 0.51 | 0.78 | 0.81 | 1.00 |
| *vp0619* | -0.28 | 0.17 | -0.31 | 0.14 | -0.27 | 0.20 | 0.78 | 0.78 | 0.78 |
| *vp0950* | 0.05 | 0.82 | -0.19 | 0.37 | -0.38 | 0.07 | 1.00 | 0.94 | 0.78 |
| *vp0952* | -0.32 | 0.12 | -0.38 | 0.06 | -0.39 | 0.05 | 0.78 | 0.78 | 0.78 |
| *vp0962* | -0.12 | 0.57 | -0.09 | 0.68 | -0.08 | 0.70 | 1.00 | 1.00 | 1.00 |
| *vp2338* | -0.15 | 0.48 | -0.21 | 0.32 | -0.13 | 0.53 | 1.00 | 0.89 | 1.00 |
| *luxS* | -0.18 | 0.40 | -0.06 | 0.78 | 0.05 | 0.83 | 0.96 | 1.00 | 1.00 |
| *flgL* | -0.13 | 0.52 | 0.13 | 0.53 | 0.32 | 0.12 | 1.00 | 1.00 | 0.78 |
| *flaA* | -0.11 | 0.61 | 0.08 | 0.72 | 0.12 | 0.55 | 1.00 | 1.00 | 1.00 |

*Means significant, *p*<0.05

**Table S9.** Spearman correlation between gene expression levels and BFI at 30 °C across all strains.

|  | *r* | *p* value | FDR-corrected *p*-values |
| --- | --- | --- | --- |
| *gefB* | 0.559^**^ | 0.003 | 0.096 |
| *cpsQ* | 0.388 | 0.050 | 0.43 |
| *tssL2* | 0.388 | 0.050 | 0.43 |
| *mfpA* | 0.380 | 0.056 | 0.43 |
| *scrA* | -0.364 | 0.068 | 0.43 |
| *gefA* | 0.343 | 0.086 | 0.46 |
| *opaR* | -0.317 | 0.115 | 0.53 |
| *ompW* | 0.289 | 0.152 | 0.55 |
| *vp0950* | 0.294 | 0.154 | 0.55 |
| *VPA1177* | -0.283 | 0.170 | 0.55 |
| *flaA* | -0.262 | 0.197 | 0.56 |
| *luxM* | -0.260 | 0.210 | 0.56 |
| *cpsA* | 0.241 | 0.235 | 0.58 |
| *flgB* | -0.212 | 0.310 | 0.71 |
| *vp0619* | -0.194 | 0.342 | 0.73 |
| *luxS* | -0.168 | 0.413 | 0.83 |
| *oxyR* | 0.120 | 0.560 | 0.90 |
| *qsvR* | 0.098 | 0.633 | 0.90 |
| *vp0962* | 0.090 | 0.663 | 0.90 |
| *gbpA* | 0.088 | 0.669 | 0.90 |
| *vp2338* | 0.086 | 0.677 | 0.90 |
| *scvE* | -0.084 | 0.682 | 0.90 |
| *vp0952* | 0.080 | 0.699 | 0.90 |
| *tnaA* | -0.078 | 0.706 | 0.90 |
| *scvO* | 0.072 | 0.727 | 0.90 |
| *tlh* | 0.060 | 0.781 | 0.93 |
| *aphA* | -0.047 | 0.822 | 0.94 |
| *scvA* | 0.031 | 0.881 | 0.95 |
| *mam7* | 0.028 | 0.892 | 0.95 |
| *flgL* | 0.015 | 0.942 | 0.97 |
| *cqsA* | 0.005 | 0.980 | 0.98 |

**Means significant, *p*<0.01


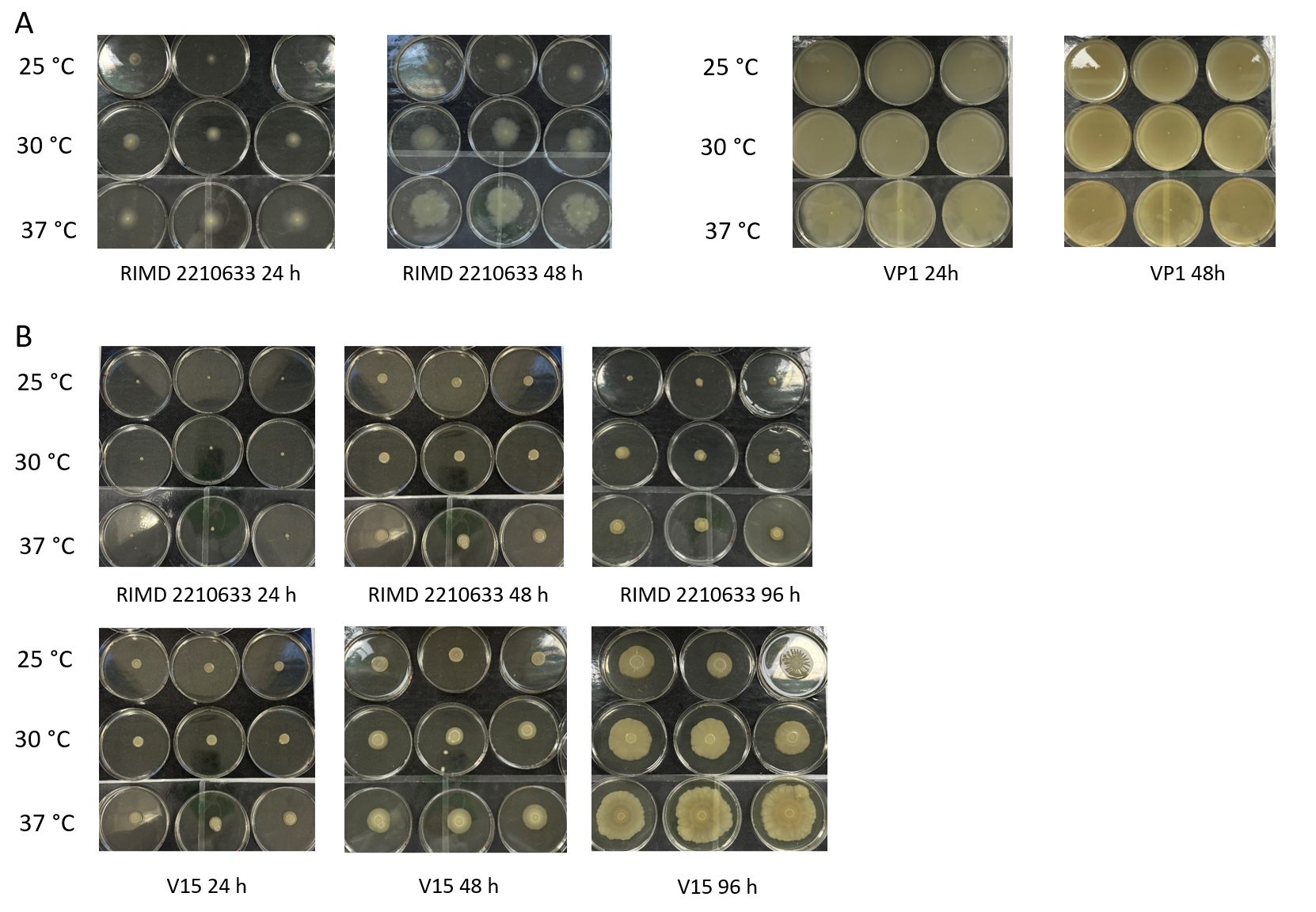


**Fig. S1.** **A)** Swimming and **B)** swarming at different temperature and time points.


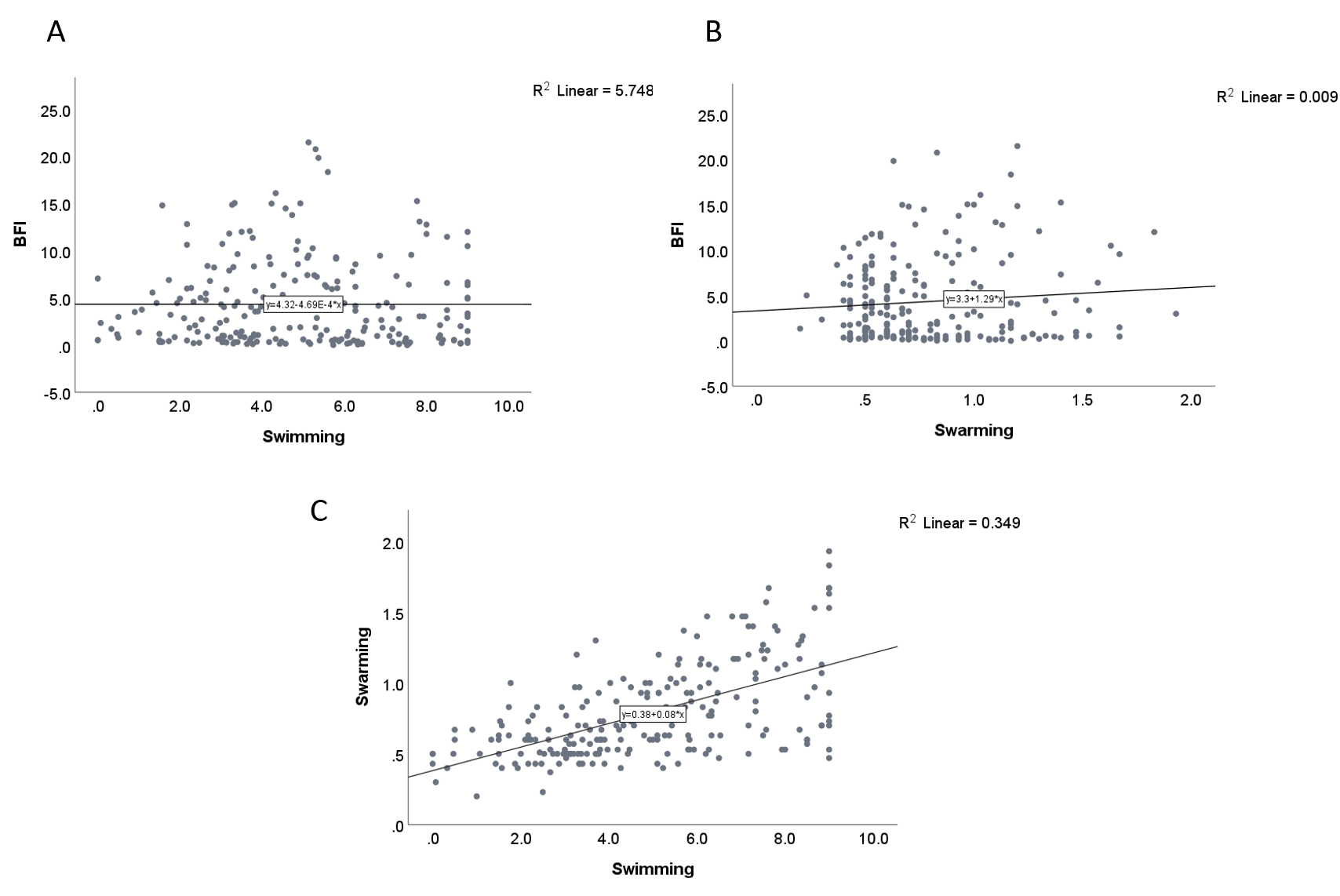


**Fig. S2.** Correlations between **A)** BFI and swimming, **B)** BFI and swarming, **C)** swimming and swarming.


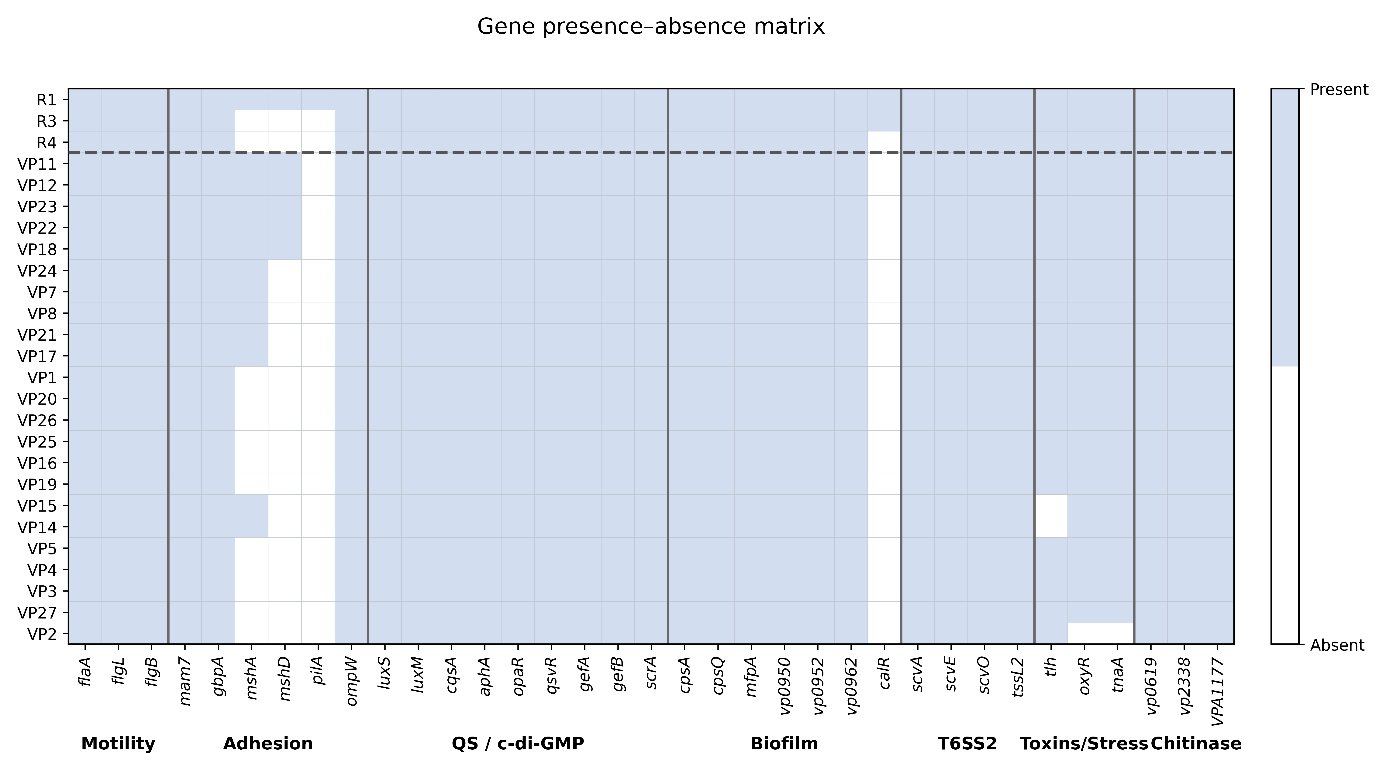


**Fig. S3.** Presence–absence matrix of biofilm- and virulence-associated genes among *V. parahaemolyticus* strains. Strains are shown by decreasing gene richness. Genes are organized into functional categories as indicated. Blue indicates gene presence; white indicates gene absence.


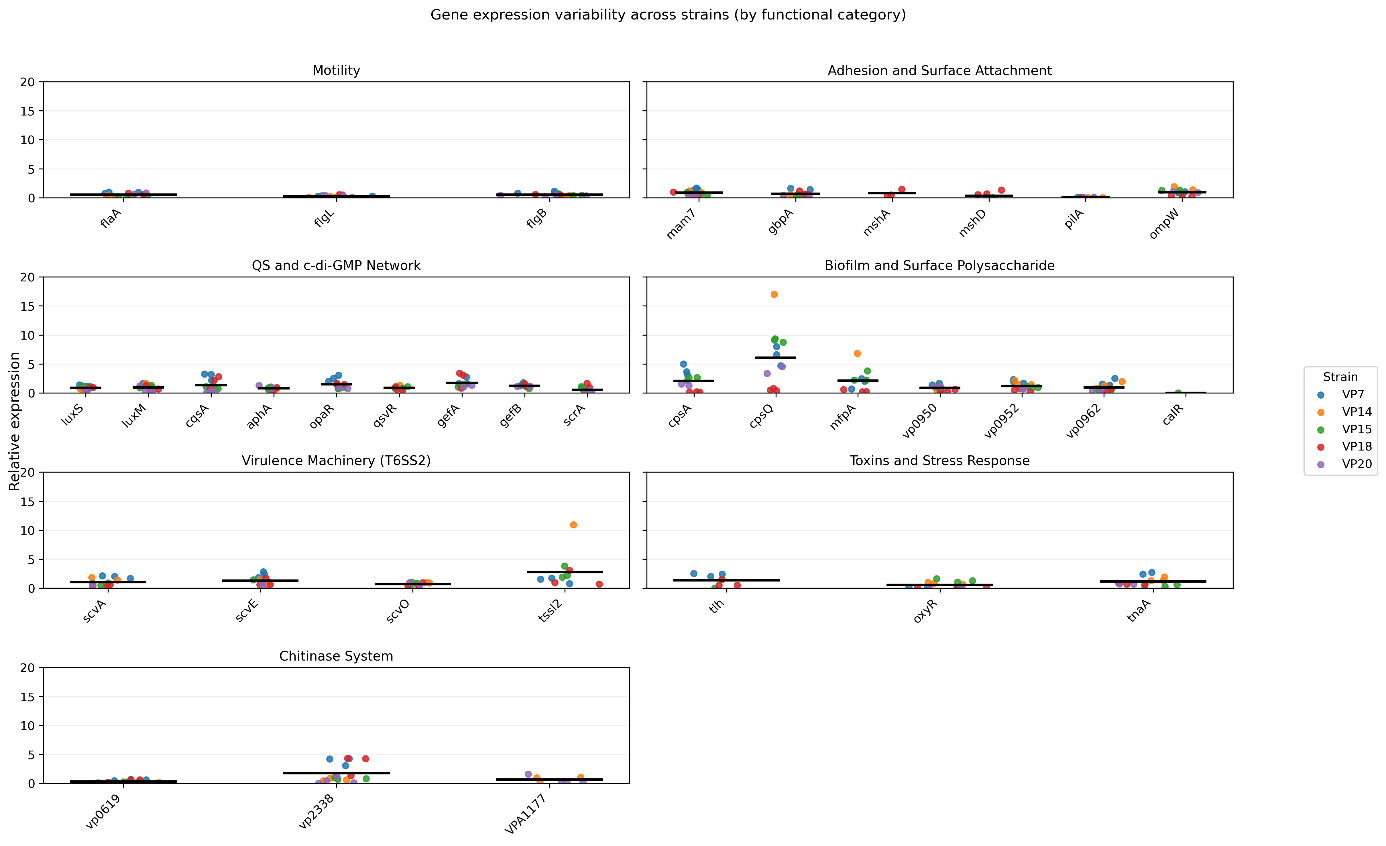


**Fig. S4**. Relative gene expression levels of selected genes measured under standardized planktonic growth conditions at 30 °C in strains exhibiting a high biofilm formation index (BFI). Genes are grouped by functional category. Horizontal bars indicate group medians. Each color represents one specific strain
